# N-Acetylcysteine Alleviates Depressive-Like Behaviors in Adolescent EAAC1^-/-^ Mice and Early Life Stress Model Rats

**DOI:** 10.7150/ijbs.97723

**Published:** 2024-10-07

**Authors:** Han-Byeol Kim, Yu-Jin Kim, Ye-Ji Lee, Ji-Young Yoo, Yoori Choi, Eun-Mee Kim, Sang Won Suh, Ran-Sook Woo

**Affiliations:** 1 Department of Anatomy and Neuroscience, College of Medicine, Eulji University, Daejeon, 34824, Republic of Korea.; 2 Department of Nuclear Medicine, Seoul National University Hospital, Seoul, 03080, Republic of Korea.; 3 Department of Paramedicine, Korea Nazarene University, Cheonan, 31172, Republic of Korea.; 4 Department of Physiology, College of Medicine, Hallym University, Chuncheon, 24252, Republic of Korea.

**Keywords:** Early life stress, Neonatal maternal separation, Excitatory amino acid carrier 1 (EAAC1), N-acetylcysteine (NAC), Depressive-like behavior, Impulsive behavior

## Abstract

Exposure to adverse experiences during early life is associated with an increased risk of psychopathology during adolescence. In a previous study, we demonstrated that neonatal maternal separation (NMS) combined with social isolation led to impulsive and depressive-like behaviors in male adolescents. Additionally, it significantly reduced the expression of excitatory amino acid carrier 1 (EAAC1) in the hippocampus. Building upon this work, we investigated the effects of N-acetylcysteine (NAC), a precursor to glutathione, in early-life stress (ELS) model rats and in EAAC1^-/-^ mice. EAAC1 plays a dual role in transporting both glutamate and cysteine into neurons. Our findings revealed that female adolescents subjected to in the ELS model also exhibited behavioral defects similar to those of males. NAC injection rescued depressive-like behaviors in both male and female NMS models, but it improved impulsive behavior only in males. Furthermore, we observed increased reactive oxidative stress (ROS) and neuroinflammation in the ventral hippocampus (vHPC) and prefrontal cortex of NMS model rats, which were mitigated by NAC treatment. Notably, NAC reversed the reduced expression of EAAC1 in the vHPC of NMS model rats. In EAAC1^-/-^ mice, severe impulsive and depressive-like behaviors were evident, and the NAC intervention improved only depressive-like behaviors. Collectively, our results suggest that ELS contributes to depression and impulsive behaviors during adolescence. Moreover, the cysteine uptake function of EAAC1 in neurons may be specifically related to depression rather than impulsive behavior.

## 1. Introduction

Early-life experiences significantly influence neural plasticity and behavioral development, particularly in the context of psychopathology. In humans, exposure to early-life adversities-such as disruption, deprivation, neglect, and abuse by caregivers-can have lasting effects on brain function throughout an individual's life, potentially increasing susceptibility to disease later in life 1, 2. Clinical studies have shown that adults who experienced parental loss during childhood are vulnerable to stress and have an elevated risk of psychiatric disorders, including depression and anxiety 3, 4. Furthermore, child abuse and neglect contribute significantly to impulsive, aggressive, and antisocial behaviors in adulthood 5, 6.

Preclinical investigations have revealed a connection between stress exposure and altered levels of glutathione (GSH) and related enzymes. Among the sodium-dependent excitatory amino acid transporters (*EAATs*) that regulate glutamate transport in the central nervous system (CNS), EAAC1 (also known as EAAT3 in humans) plays a crucial role. Excitatory amino acid carrier 1 (EAAC1) facilitates the transport of cysteine into neurons, which serves as the rate-limiting substrate for neuronal synthesis of GSH 7, 8.

Notably, EAAC1 plays a crucial role in facilitating cysteine intake within neurons rather than primarily participating in glutamate clearance from the brain's extracellular space 9. *EAAC1 expression is particularly robust* in the hippocampus (HPC), where it is present at 2- to 3-fold higher levels than that in other brain regions 10. In rodents, the ventral hippocampus (vHPC), which is analogous to the human anterior region, plays significant roles in the stress response and anxiety. Conversely, the dorsal hippocampus (dHPC), akin to the posterior regions of the human HPC, is preferentially involved in spatial learning and memory 11-13. In our study, we investigated the biochemical changes induced by early-life stress (ELS) in adolescent male and female rats, with a focus on the vHPC and prefrontal cortex. Reactive oxidative species (ROS), highly reactive ions or free radicals containing oxygen, disrupt redox homeostasis and cellular function, leading to neuronal damage in the brain. EAAC1 is closely associated with oxidative stress in neurons because of its role in GSH production, a critical factor for mitigating oxidative damage 14. Furthermore, EAAC1-deficient mice present decreased GSH levels, increased oxidative stress in the cortex and HPC, and cognitive impairment 15.

Neonatal maternal separation (NMS) serves as a widely used animal model of ELS. Our previous research showed that when NMS is combined with social isolation, it triggers impulsive and depressive-like behaviors in male adolescents. Interestingly, EAAC1 knockout (EAAC1^-/-^) mice exhibit similar behavioral patterns 16. Building upon these findings, we sought to investigate whether the reduction in cysteine uptake, which is attributed to EAAC1, affects the amelioration of impulsive and depression-like behaviors in both male and female adolescent NMS rats. Our observations confirmed that both male and female NMS model animals displayed depression-like behaviors and impulsivity due to early-life stress. In this study, we administered N-acetylcysteine (NAC) injections to both male and female adolescent NMS model animals. Our findings revealed a significant improvement in the male subjects following the NAC injection, whereas the female subjects did not exhibit the same level of improvement. The beneficial effects observed in male subjects can be attributed to the reductions in ROS levels and neuroinflammation in the NMS models, which were achieved through the NAC intervention. Furthermore, we observed a restoration of EAAC1 expression, which had previously been diminished in the NMS model. This restoration also played a significant role in the observed recovery effects.

In addition to our previous investigations, we extended our study to examine behaviors in adolescent EAAC1^-/-^ mice after the administration of NAC. Our results revealed a significant recovery of depression-like behaviors; however, no noticeable change in impulsive behaviors was observed. Taken together, these findings underscore the pivotal role of the cysteine uptake function of EAAC1 in alleviating depression-like behaviors but not in modulating impulsive behaviors during adolescence. These findings suggest the potential specificity of the therapeutic effects of EAAC1-targeted interventions. These findings highlight the importance of understanding the distinct roles of EAAC1 in different behavioral phenotypes, which could guide the development of more targeted and effective therapeutic strategies. This study is a significant step forward in our understanding of how ELS can impact behavior and the potential therapeutic strategies that can be employed.

## 2. Materials and methods

### 2.1. Reagents and antibodies

NAC (A9165) was purchased from Sigma-Aldrich (St. Louis, MO, USA). Dihydroethidium (DHE) (D11347) was purchased from Invitrogen by Thermo Fisher Scientific (Eugene, OR, USA). Antibodies were obtained from Millipore Corporation (Chemicon, MA, USA; EAAC1, MAB1587), Cell Signaling Technology (MA, USA; EAAC1, 12179), Santa Cruz Biotechnology Inc. (Santa Cruz, CA, USA; gp91^phox^, sc-130545; β-actin, sc-47778), Proteintech (Rosemont, USA; iNOS, 18985-1-AP), Novus Biologicals (Centennial, CO, USA; AIF-1/Iba1, NB100-1028), R&D Systems (Minneapolis, USA; 4-hydroxynonenal, MAB3249), and Abcam (Cambridge, UK; GFAP, ab7260).

### 2.2. Animals and maternal separation procedure

Pregnant Sprague-Dawley (SD) rats at 15-17 days of gestation were obtained from a laboratory animal supplier (Samtako bio Korea) and were housed individually in cages under standard laboratory conditions with a 12-h light / 12-h dark cycle. Experiments with animals were approved by the Institutional Animal Care and Use Committee of Eulji University (EUIACUC 22-20). On postnatal day (PND) 1, the litters were culled to 6-10 pups at a constant sex ratio of 1:1 to avoid sex-based maternal behavioral biases. During separation sessions, the pups were removed from the mother and separated individually for periods of 3 hrs per day (PND 2-21). Beginning on PND 1, the pups were weighed daily, and their overall health was assessed (i.e., milk visible in the pup's stomach and pups were age-appropriately active). The male and female animals were randomly allocated to the following four groups: saline, NAC, NMS, and NMS + NAC. EAAC1^-/-^ mice are also housed in a regulated environment. EAAC1^-/-^ mice were descendants of the strain established by Peghinni *et al.* 17, in which exon 1 is disrupted by a neomycin resistance cassette. EAAC1^-/-^ mice were outbred to wild-type (WT) C57BL/6 mice for more than 10 generations prior to these studies. A WT colony was maintained using the WT offspring from the latter outcrosses.

### 2.3. Behavioral analysis

The behavior of the animals was investigated in parallel. All behavioral tests were conducted during the same phase of the circadian cycle (i.e., the light phase) to minimize the influence of circadian rhythms. One investigator performed all the behavioral tests to prevent interobserver variability due to differences in the handling of the pups. The exploratory and depressive-like behaviors of the animals were tested during adolescence.

#### 2.3.1. Exploratory behavior in the open field test

The open field test (OFT) was performed as previously described 16, 18. The open field-test arena (rat: 60 cm × 60 cm × 30 cm; mouse:30 cm × 30 cm × 25 cm) was divided into 25 squares using a computer tracking system (SMART® version 3.0.05; Panlab Harvard Apparatus, Barcelona, Spain). The nine squares in the central area were defined as the inner zone, the four squares at each corner were defined as the edge zone, and the remaining squares in the periphery and the edge zone were defined as the side zone. Adolescent rats (males: PND 38, females: PND 33) and EAACl^ -/-^ (males: PND 38) mice were allowed to explore the testing arena freely for 5 min. The running distance of the rats in the edge, side, and inner zones, as well as the total distance traveled, the time spent, the zone transition number, and the velocity, were quantified. Additionally, the occurrence and duration of wall rearing, stretching, and grooming behaviors were analyzed.

#### 2.3.2. Elevated plus maze test

The elevated plus maze (EPM) test was used to observe anxiety-like behaviors in rats and mice 19. The plus-shaped maze apparatus consisted of four arms: two open arms and two closed arms. For the rats, the maze height was 72 cm above the floor, with each arm being 10 cm wide and 100 cm in length. For the mice, the maze height was 40 cm above the floor, with each arm being 6 cm wide and 65 cm in length. During the EPM test, the animals (males: PND 39, females: PND 34) were placed in the maze for 5 min, and their locomotor behavior was automatically tracked. The anxiety-like behavior of each animal was measured by analyzing the video recording, which included the time spent in the open/closed arms, number of entries, and distance traveled. The apparatus was thoroughly cleaned with 70% ethanol between sessions to prevent contamination.

#### 2.3.3. Cliff avoidance test (CAT)

The CAT was used to observe impulsive behavior in animals 20, 21. The CAT prototype platform measured 15 cm in diameter and 25 cm in height. It was divided into two zones: a central (inner) zone with a diameter of 8 cm and a surrounding border zone measuring 3.5 cm in width. At the start of the test, the animals (males: PND 39, females: PND 34) were placed in the central zone of the platform. The experiment lasted 10 minutes, during which the time the animals spent in each zone and the instances of the animals falling from the platform to the floor were measured and analyzed.

#### 2.3.4. Visual cliff avoidance test (VCAT)

The VCAT was used to observe impulsive behaviors in animals 22, 23. The animals were tested in an open acrylic box (60 cm × 60 cm × 40 cm; thickness: 0.8 cm) placed at the edge of a table, 70 cm above the floor. The box had a transparent bottom and was positioned so that half of it rested on the table (table zone), whereas the other half was suspended above the floor (cliff zone). A black and white checkered board (60 cm × 45 cm; checker size: 4 cm × 4 cm) was placed on both the table and the floor to emphasize the cliff drop-off. The animals (males: PND 40, females: PND 35) were placed on a “stage” (an acrylic box: 17.5 cm × 26 cm × 8 cm) positioned in the center of the main acrylic box. The experiment lasted 6 minutes, during which the time the animals spent with their front paws in the cliff zone was measured and compared.

#### 2.3.5. Tail suspension test (TST)

The test was performed as previously described 24. The animals (males: PND 40, females: PND 35) were individually suspended by the tails; climb stoppers were placed around their tails before applying adhesive tape to prohibit climbing. The duration of immobility was measured for 6 min and was defined as the time when the mice were completely motionless and hung passively.

The animals (males: PND 40, females: PND 35) were individually suspended by the tails; climb stoppers were placed around their tails before applying adhesive tape to prohibit climbing. The duration of immobility was measured for 6 min and was defined as the time when the mice were completely motionless and hung passively. The animals (males: PND 40, females: PND 35) were individually suspended by the tails; climb stoppers were placed around their tails before applying adhesive tape to prohibit climbing. The duration of immobility was measured for 6 min and was defined as the time when the mice were completely motionless and hung passively.

#### 2.3.6. Forced swimming test (FST)

The FST was conducted as described previously 25. After the TST, the same animals were subjected to the FST (males: PND 41-42; females: PND 36-37). Each rat was allowed to swim in an acrylic cylinder (20 cm diameter; 45 cm height) filled with water to a depth of 30 cm (23-25 °C). The animals were individually placed into the cylinder containing water for 15 min as preswim trials, and then they were carefully dried and returned to their home cage. The following day, the animals were subjected to the FST for 5 min (test trial), and the immobility and climbing times were recorded in seconds. Between every animal, the water was removed, and the mixture was refilled again to avoid any odor cues. Each animal was allowed to swim in an acrylic cylinder (10 cm diameter; 45 cm height) filled with water to a depth of 15 cm.

#### 2.3.7. Sucrose preference test (SPT)

As a core component of depression, anhedonia was assessed through a sucrose preference test (males: PND 36-42; females: PND 31-37) 26. First, the animals were individually placed in cages and acclimated to two bottles containing a 1% sucrose solution. The animals were presented with a 1% sucrose solution for 24 hrs, the drinking water was replaced, and their positions were changed after 12 hrs. The animals were subsequently deprived of food and water for 24 hrs before the test day, and free access was provided to two identical bottles containing 1% sucrose and normal drinking water from 08:00 to 10:00. Finally, the consumption of the sucrose solution and drinking water and the sucrose preference (%) were measured based on the percentage of sucrose consumption relative to the sum of sucrose and water consumption. The animals were acclimated for 12 h to a 1% sucrose solution. After 12 hrs of food and water deprivation, the mice were given one bottle of l% sucrose water and one bottle of pure water, and two bottles of liquid were removed 2 hrs later and weighed. Sucrose preference (%) was defined as [sucrose intake (g)/(sucrose intake + water intake) (g)] × 100.

### 2.4. Immunofluorescence analysis

Immunofluorescence staining of rat brain sections was performed as previously described. Briefly, cryosections (15 µm) were fixed with 4% paraformaldehyde in phosphate-buffered saline** (**PBS) for 20 min. After fixation, the sections were permeabilized for 10 min with 0.5% Triton X-100 in PBS. The sections were incubated overnight at 4 °C with PBS containing rabbit anti-iNOS (1:100), rabbit anti-GFAP (1:100), mouse EAAC1 (1:100), mouse 4-HNE (1:500) and goat anti-Iba1 (1:100) antibodies, followed by an incubation with Alexa Fluor® 488-conjugated rabbit or mouse anti-goat IgG and Alexa Fluor 594-conjugated goat anti-rabbit IgG or donkey anti-goat IgG secondary antibodies (Jackson ImmunoResearch Laboratories, Inc., 1:400) in buffer for 2 hrs at room temperature. The nuclei were counterstained with 4',6-diamidino-2-phenylindole (DAPI; 10 μM in PBS) for 5 min. The stained cells were mounted in Vectorshield (Vector Laboratories) and observed under an LSM 880 confocal microscope with Airyscan (Carl Zeiss AG, Oberkochen, Germany).

### 2.5. Immunohistochemistry

Dewaxed sections were boiled in 0.1 mol/L citrate-buffered saline (pH 6.0) for 10 min to retrieve antigens. After cooling for 30 min, the sections were rinsed with PBS. After fixation, endogenous peroxidase activity was quenched by the addition of 1% hydrogen peroxide in 10% methanol and an incubation for 30 min. After two washes with PBS-T (0.2% Triton X-100 in 0.1 mol/L PBS, pH 7.6) for 5 min each, the sections were blocked for 1 hr in blocking solution (5% host serum + 1% BSA in PBS-T) and incubated with the primary antibody (anti-EAAC1, 1:200) at 4 °C overnight. After washes with PBS-T, the sections were incubated with a biotinylated secondary antibody for 1 hr at RT. After rinses with PBS and treatment with an avidin-biotin-peroxidase complex (Vectastain Elite ABC kit) for 1 hr at RT, the sections were developed for 5 min in a 0.05% DAB solution. The stained cells were mounted in Vectorshield (Vector Laboratories) and observed under an LSM 880 confocal microscope with Airyscan (Carl Zeiss AG, Oberkochen, Germany).

### 2.6. Dihydroethidium (DHE) staining

The brain was immediately frozen in embedding medium to assess superoxide production. Briefly, fixed cryosections (15 µm) were incubated with DPBS containing 10 μM DHE (Invitrogen CA, USA) for 30 min at 37 °C in a dark room. The sections were then washed three times with DPBS and mounted in Vectorshield (Vector Laboratories). Fluorescence images were acquired with an LSM 880 confocal microscope with Airyscan (Carl Zeiss AG, Oberkochen, Germany). Images were obtained at an excitation wavelength of 561 nm and an emission wavelength of 640 nm.

### 2.7. Assays of inflammatory factor contents

Neuroinflammation was assessed by measuring the levels of inflammatory cytokines in the rat hippocampus. Interleukin-6 (IL-6), interleukin-1β (IL-1β), and TNFα levels were measured using commercially available ELISA kits (R&D Systems, Minneapolis, MN; IL-6: DY501; IL-1β: DY506; TNFα: DY510) according to the manufacturer's instructions.

### 2.8. Glutathione peroxidase (GPx) activity assay

GPx activity was determined using a Cayman glutathione peroxidase assay kit (Cayman Chemical Company, MI, USA) according to the manufacturer's protocol. The tissues were homogenized on ice in cold assay buffer and then centrifuged at 10,000 × g for 15 minutes at 4 °C. Next, 50 μl of the supernatant was added to a 96-well plate with 50 μl of assay buffer. The reaction mixture was added to each sample and incubated for 15 min to deplete all the GSSG in the samples. Ten microliters of cumene hydroperoxide substrate was subsequently added to initiate the enzymatic reaction. The absorbance was immediately measured at a wavelength of 340 nm using a VICTOR X3 multilabel plate reader (PerkinElmer, Shelton, USA). GPx activity was calculated from an NADPH standard curve.

### 2.9. Superoxide dismutase (SOD) activity assay

SOD activity was measured using a commercially available kit (Cayman Chemical Company, MI, USA) according to the manufacturer's protocol. The tissues were homogenized in cold 20 mM HEPES buffer (pH 7.2) and centrifuged at 1,500 × g for 5 min at 4 °C. Each sample (10 μl) was added to a 96-well plate with 200 μl of the diluted radical detector. Then, 20 μl of diluted xanthine oxidase was added to initiate the enzymatic reaction. The absorbance was immediately measured at a wavelength of 450 nm using a VICTOR X3 multilabel plate reader.

### 2.10. Western blot analysis

Western blotting was performed as previously described 27. Briefly, the tissues were homogenized using a modified homogenization buffer (50 mM Tris-HCl [pH 7.4], 150 mM NaCl, 1% NP-40, 0.25% sodium deoxycholate, 1 mM PMSF, 1 mM EDTA, and 1 μg/ml each of the protease inhibitors aprotinin, leupeptin, and pepstatin). The samples were then resolved on SDS‒PAGE gels and transferred to nitrocellulose membranes that were subsequently blocked with Tris-buffered saline (TBS) containing 5% fat-free milk and 0.05% Tween-20 for 1 hr. Next, the membranes were incubated overnight at 4 °C with primary antibodies (anti-EAAC1 (1:1,000), anti-iNOS (1:1,000), anti-gp91^phox^ (1:1,000), and anti-β-actin (1:5,000)) and developed with horseradish peroxidase-conjugated secondary antibodies. Immunodetection was performed with a chemiluminescence system (Amersham Pharmacia) and a ChemiDoc^TM^ tough imaging system (Bio-Rad, California, USA).

### 2.11. Statistical analysis

The data are presented as the means ± SEMs of three or more independent experiments. Statistical analysis was performed with Graph Pad Prism 9 (version 9.4.1, GraphPad Prism software, San Diego, California, USA). The normality of the distribution was tested using the Shapiro-Wilk, Anderson-Darling, D'Agostino and Pearson, and Kolmogorov-Smirnov tests. Nonnormally distributed data were analyzed using the Kruskal‒Wallis test and one-way ANOVA. For multiple comparisons of significant results via one-way and two-way ANOVA, we employed the Tukey test or Dunn's method. A value of *P* < 0.05 was considered statistically significant.

## 3. Results

### 3.1. NAC rescued impulsive-like behaviors in the adolescent male NMS group in the OFT but not in the female NMS group

**Figure 1:**
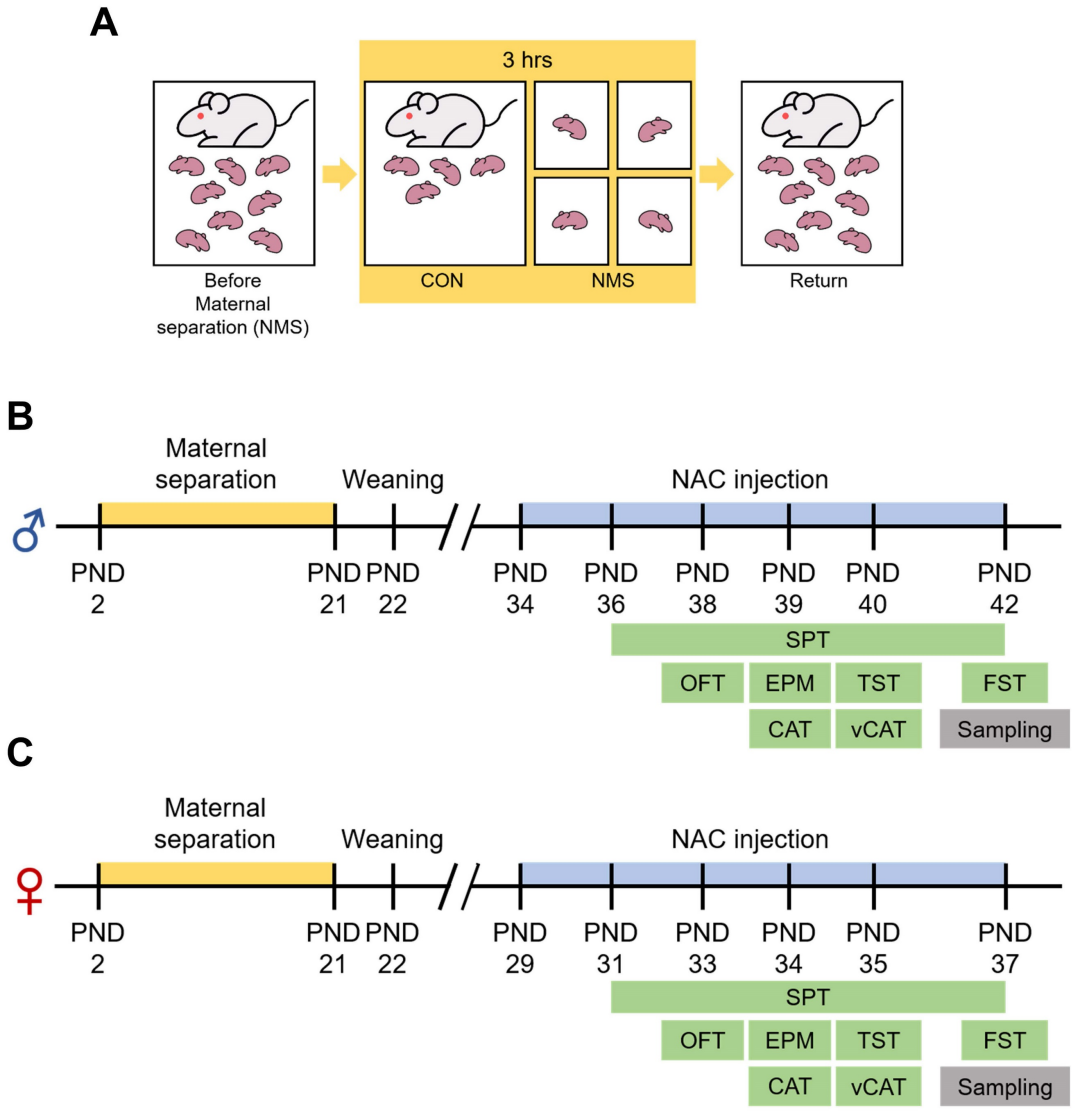
** Experimental procedure and schedule for the NAC injection and behavioral tests.** (A) A schematic diagram of the experimental procedure. Pups were individually separated from their mothers and littermates for a duration of 3 hrs/day, from 10:00 to 13:00. This maternal separation was applied to the experimental subjects from PND 2 to PND 21. Behavioral experiments were conducted from PND 36 to PND 42 in male rats (B) and from PND 31 to PND 37 in female rats (C). The i.p. injection of NAC commenced 2 days prior to the behavioral tests.

**Figure 2:**
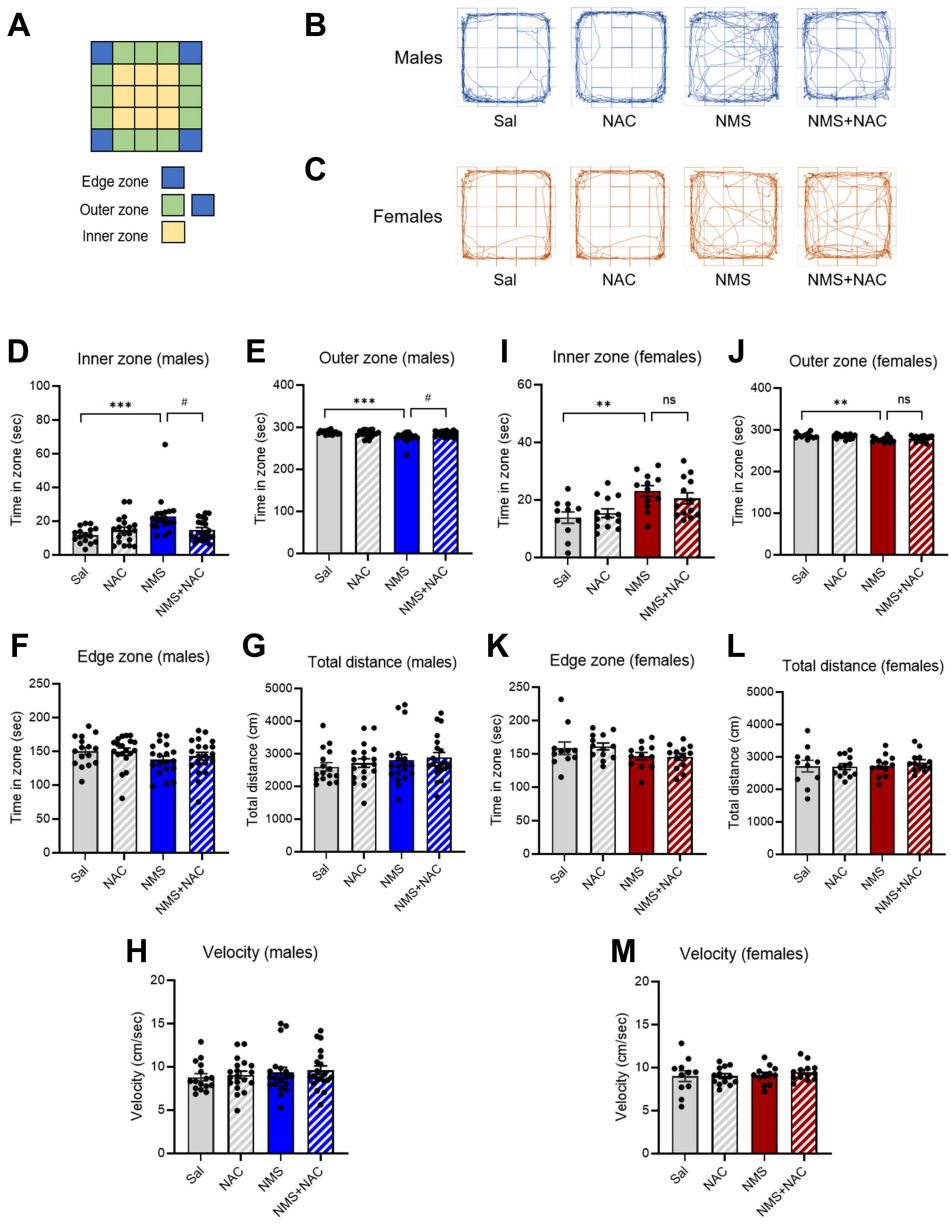
** NAC mitigates NMS-induced impulsive behaviors in male rats in the OFT.** (A) A schematic of the tested open field arena, displaying the edge, outer, and inner zones. Representative tracks of male (B) and female (C) rats in the Sal, NAC, NMS, and NMS+NAC groups. The bar graph shows the time spent in the inner zone (D), outer zone (E), and edge zone (F) by male rats. The bar graph shows the total distance traveled (G) and velocity (H) of male rats. The bar graph shows the time spent in the inner zone (I), outer zone (J), and edge zone (K) by female rats. The bar graph shows the total distance (L) and velocity (M) of female rats. The locomotor behavioral data are presented as the means ± S.E.M.s. Differences among the experimental groups were determined using one-way ANOVA with Tukey's multiple comparisons test. **p < 0.01, ***p < 0.001 and ^#^*p* < 0.05.

The significance of the consequences of ELS on humans is increasingly recognized, leading to the extensive use of NMS as an ELS animal model. In this study, we employed an NMS model combined with social isolation (SI), involving daily separation of pups from their mothers and siblings for 3 hrs (Figure 1A). We conducted behavioral studies using animals of both genders during adolescence (Figure 1B and C). The open field apparatus, a square arena (5×5), is divided into edge, outer, and inner zones (Figure 2A). Compared with those in the Sal group, both the male and female NMS groups exhibited significant increases in impulsive-like behaviors. The male NMS group demonstrated greater exploration of the inner zone than the Sal group (Sal group, 11.78 ± 1.11; n = 16; NMS group, 22.83 ± 2.58; n = 19; *** p < 0.001; Figure 2B and D), and the female NMS group also showed greater exploration of the inner zone (Sal group, 13.85 ± 1.87; n = 11; NMS group, 23.12 ± 1.76; n = 12; ** p < 0.01; Figure 2C and I). The rats were treated with NAC (50 mg/kg, i.p.) to assess its effects on NMS-induced impulsive-like behaviors (Figure 1B and C). NAC treatment significantly mitigated the increase in impulsive-like behaviors observed in the open field in the male NMS group (NMS group, 22.83 ± 2.58; n = 19; NMS + NAC group, 14.85 ± 1.35; n = 20; ^#^*p* < 0.05; Figure 2B and D) but not in the female NMS group (Figure 2C and I). Parameters such as the total distance traveled and velocity of movement were comparable between the Sal and NMS groups, indicating that neither the male nor the female NMS groups of adolescent rats exhibited impaired general motor ability (*p* > 0.05; Figure 2G-H and 2L-M).

### 3.2. NAC alleviated impulsive-like behaviors in the adolescent male NMS group in the EPM test but not in the female NMS group

**Figure 3:**
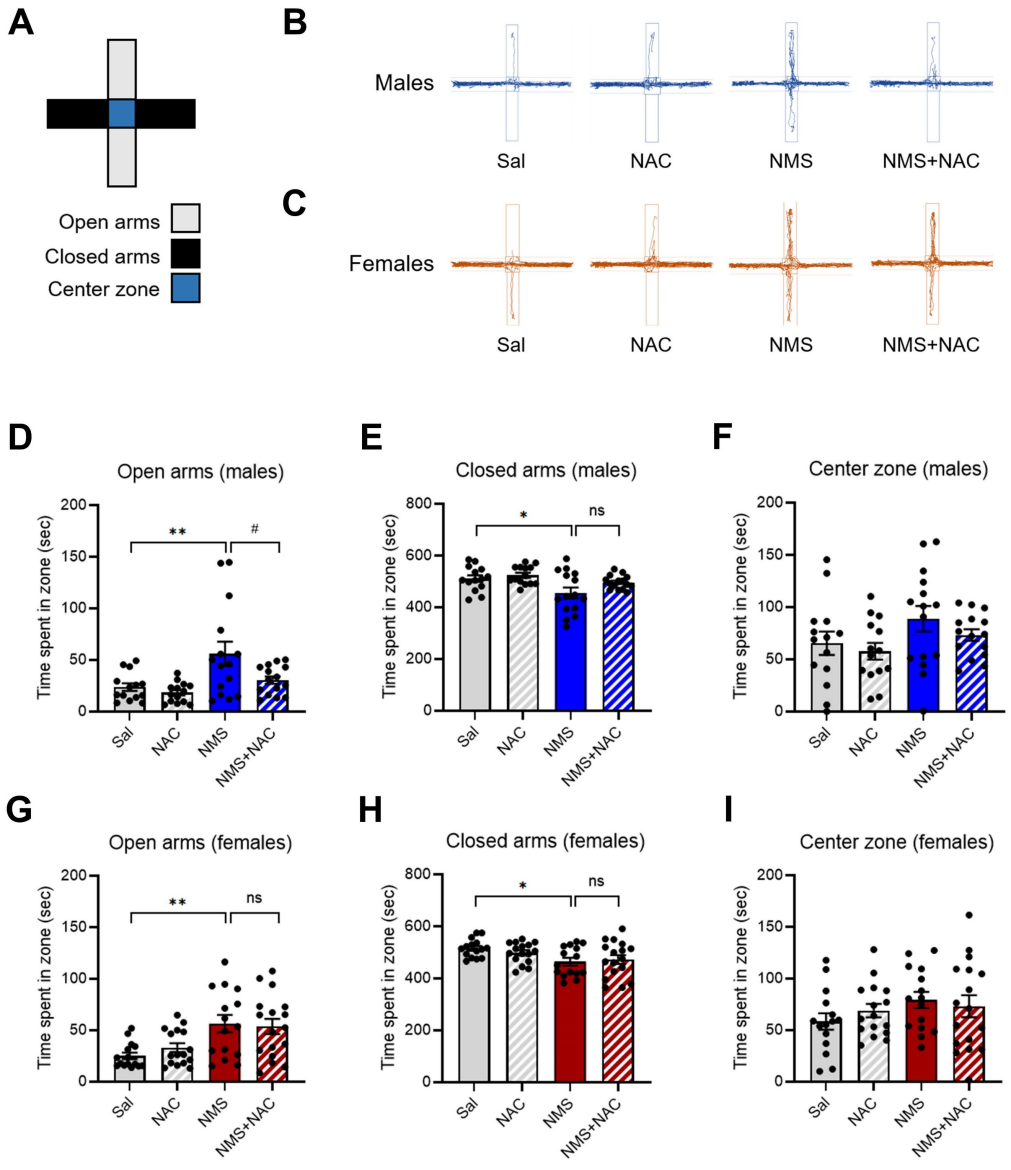
** NAC rescues NMS-induced impulsive behaviors in male rats in the EPM.** (A) A schematic of the EPM, displaying the open, closed, and center zones. Representative tracks of male (B) and female (C) rats in the Sal, NAC, NMS, and NMS+NAC groups. The bar graph shows the time spent in the open arms (D), closed arms (E), and center zone (F) by male rats. The bar graph shows the time spent in the open arms (G), closed arms (H), and center zone (I) by female rats. The quantified data are presented as the means ± S.E.M.s. Differences among the experimental groups were determined using one-way ANOVA with Tukey's multiple comparisons test. *p < 0.05, **p < 0.01, and ^#^*p* < 0.05.

We conducted an EPM test using both the Sal and NMS groups to validate the impulsive-like behaviors (Figure 3A). An increase in the percentage of time spent in the open arms was interpreted as a sign of impulsive-like behavior, as previously described 28, 29. Compared with the Sal group, both the male and female NMS groups presented increased impulsive-like behaviors when multiple parameters (such as time spent in the open arms, closed arms, and center zone) were compared. The male NMS group spent significantly more time in the open arms than did the Sal group (Sal group, 23.75 ± 3.57; n = 14; NMS group, 56.05 ± 11.24; n = 15; **p < 0.01; Figure 3B and D), and the female NMS group also spent significantly more time in the open arms (Sal group, 25.06 ± 3.07; n = 15; NMS group, 56.40 ± 8.17; n = 15; **p < 0.01; Figure 3C and G). Treatment with NAC significantly mitigated the increase in impulsive-like behaviors observed in the open arms in the NMS group (NMS group, 56.05 ± 11.24; n = 15; NMS + NAC group, 30.54 ± 3.46; n = 15; ^#^*p* < 0.05; Figure 3B and D). However, NAC treatment did not alleviate the impulsive-like behaviors observed in the female NMS group. These results are consistent with those obtained using the OFT.

### 3.3. NAC alleviated impulsive-like behaviors in the adolescent male NMS group in the CAT test but not in the female NMS group

**Figure 4:**
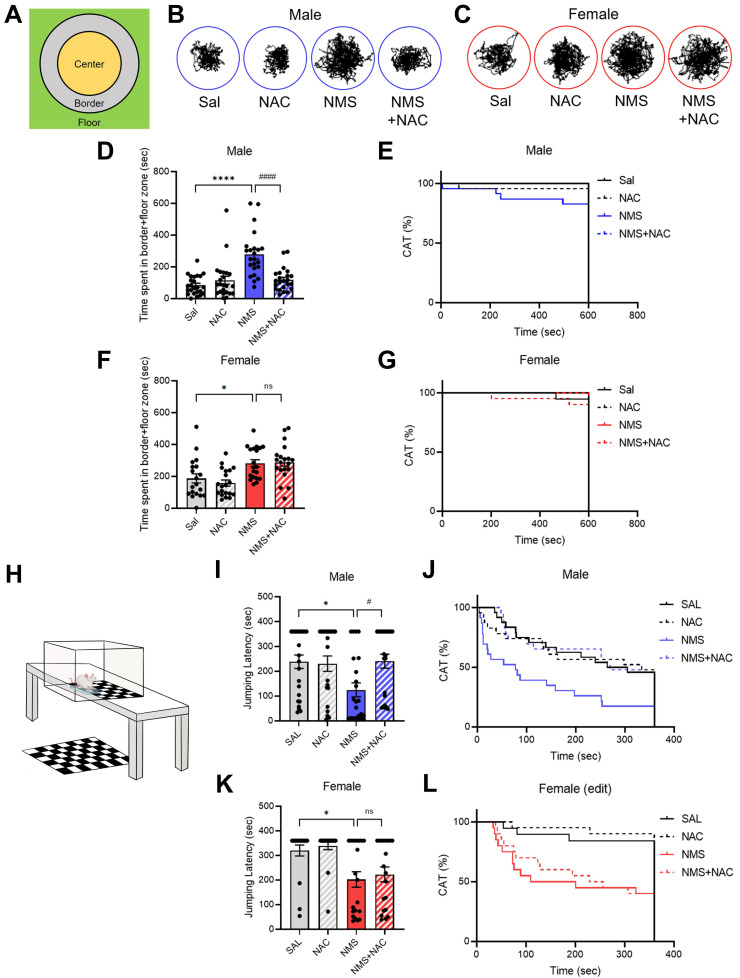
** NAC rescues NMS-induced impulsive behaviors in male rats in the VCAT.** (A) Schematic representation of the test arena in the CAT, highlighting the center, border, and floor zones. (B) Representative tracks of male rats treated with Sal, NAC, NMS, or NMS+NAC. (C) Representative tracks of female rats treated with Sal, NAC, NMS, or NMS+NAC. (D) Bar graph showing the time spent in the border + floor zones. (E) Bar graph showing the CAT (%) in male rats. (F) Bar graph showing the time spent in the border + floor zones by female rats. (G) Bar graph showing the CAT (%) in female rats. (H) Schematic of the VCAT platform. (I) Bar graph showing the jumping latency of male rats. (J) Bar graph showing the CAT activity (%) of male rats (CON, NMS: chi square = 7.413, ***p < 0.01; NMS, NMS+NAC: chi square = 7.674, ^##^*p* < 0.01). (K) Bar graph showing the jumping latency of female rats. (L) Bar graph showing the CAT activity (%) of female rats (CON, NMS: chi square = 9.061, ***p < 0.001). The data quantifying impulsive-like behaviors are presented as the means ± S.E.M.s. Differences in the experimental groups were determined using one-way ANOVA with Tukey's multiple comparisons test. *p < 0.05, ****p < 0.0001, ^#^*p* < 0.05 and ^####^*p* < 0.0001.

We conducted a CAT test on both the Sal and NMS groups to validate the impulsive-like behaviors (Figure 4A-G). We did not observe a significant difference in falling from the platform to the floor between the male NMS group and the female NMS group (Figure 4E and G). However, both the male and female NMS groups exhibited increased impulsive-like behaviors compared with the Sal group, as evidenced by the time spent in the border + floor zone (males: Sal group, 84.73 ± 11.82; n = 24; NMS group, 278.89 ± 28.68; n = 23; females: Sal group, 187.41 ± 27.96; n = 19; NMS group, 283.09 ± 21.68; n = 20; *p < 0.05 and ****p < 0.0001; Figure 4B-D and F). Treatment with NAC significantly mitigated the decrease in impulsive-like behaviors observed in the border + floor zone in the male NMS group (NMS group, 278.89 ± 28.68; n = 23; NMS + NAC group, 119.68 ± 14.45; n = 23; ^####^*p* < 0.0001; Figure 4B and D). However, NAC treatment did not alleviate the impulsive-like behaviors observed in the female NMS group (Figure 4C and F). These results are consistent with those observed in the OFT and EPM.

### 3.4. NAC alleviated impulsive-like behavior in the adolescent male NMS group in the VCAT test but not in the female NMS group

We further confirmed the impulsive behaviors by conducting the VCAT test (Figure 4H-L). Compared with those in the Sal group, both the male and female NMS groups presented increased impulsive behaviors, as evidenced by the time taken to reach the cliff zone. The male NMS group took significantly less time to reach the cliff zone than the Sal group did (males: Sal group, 238.47 ± 26.44; n = 24; NMS group, 124.97 ± 27.36; n = 23; females: Sal group, 320.15 ± 21.75; n = 19; NMS group, 201.43 ± 32.21; n = 20; *p < 0.05; Figure 4I-L). Treatment with NAC significantly mitigated the decrease in jumping latency in the male NMS group (NMS group, 124.97 ± 27.36; n = 23; NMS+NAC group, 241.01 ± 27.51; n = 23; ^#^*p* < 0.05; Figure 4I and J). However, NAC treatment did not alleviate the impulsive-like behaviors observed in the female NMS group (Figure 4K and L). These results are consistent with those observed in the OFT, EPM, and CAT.

### 3.5. NAC alleviated depressive-like behaviors in both the male and female adolescent NMS groups in the TST and FST

**Figure 5:**
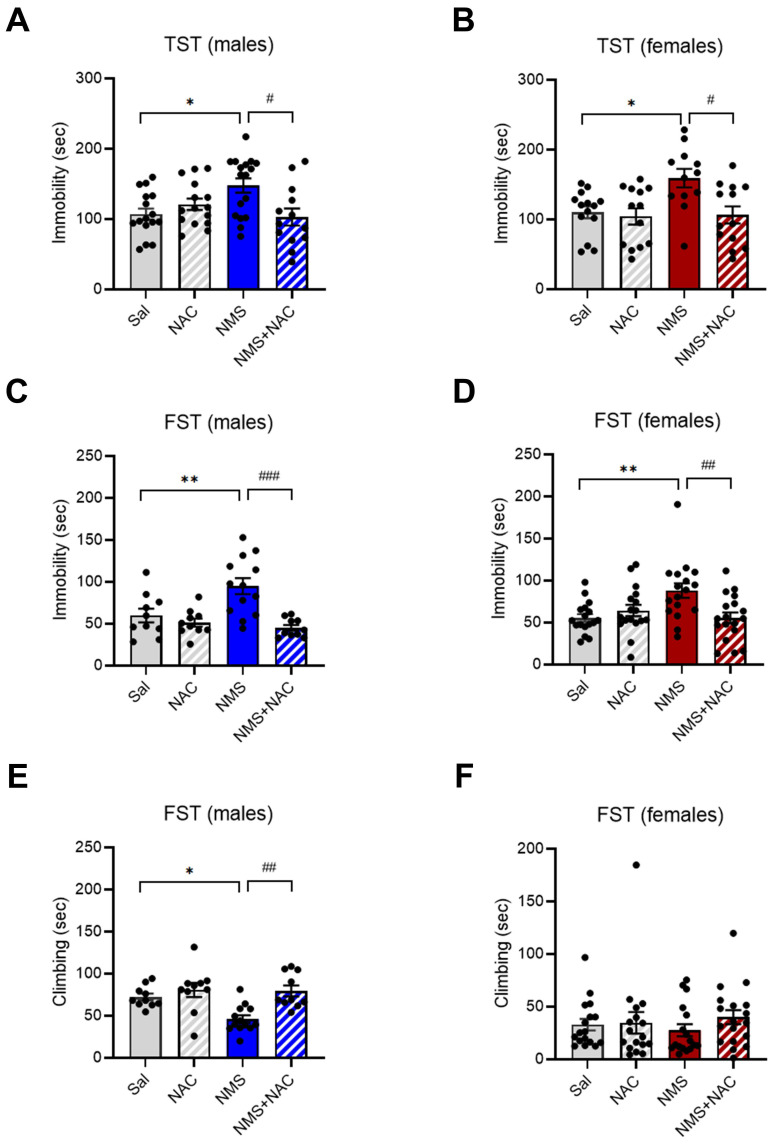
** NAC rescues NMS-induced depressive-like behaviors in male and female rats.** NMS rats were tested in the TST, and the immobility time was measured. The bar graph shows the immobility time in of male rats (A) and female rats (B). NMS rats were also tested in the FST. The bar graphs show the time spent immobile (C) and climbing (E) by male rats and the time spent immobile (D) and climbing (F) by female rats. The data quantifying depressive-like behaviors are presented as the means ± S.E.M.s. Differences in the experimental groups were determined using one-way ANOVA with Tukey's multiple comparisons test. *p < 0.05, **p < 0.01, ^#^*p* < 0.05, ^##^*p* < 0.01, and ^###^*p* < 0.001.

We further analyzed immobility and climbing times using the TST and FST, which measure despair and depressive-like behaviors, respectively. We found that NMS induced depressive-like behaviors in both male and female adolescents. Both the male and female NMS groups presented a significant increase in the duration of immobility during the TST (males: Sal group, 106.96 ± 7.70; n = 16; NMS group, 147.77 ± 9.81; n = 17; females: Sal group, 110.23 ± 8.28; n = 14; NMS group, 159.10 ± 12.59; n = 12; *p < 0.05; Figure 5A and B). Treatment with NAC significantly rescued the increase in the duration of immobility observed in both the male and female NMS groups during the TST (males: NMS group, 147.77 ± 9.81; n = 17; NMS + NAC group, 102.96 ± 11.60; n = 13; females: NMS group, 159.10 ± 12.59; n = 12; NMS + NAC group, 106.49 ± 11.77; n = 13; ^#^*p* < 0.05; Figure 5A and B). Both the male and female NMS groups also presented a significant increase in the duration of immobility during the FST (males: Sal group, 59.87 ± 7.42, n = 10; NMS group, 94.83 ± 9.19, n = 13; females: Sal group, 55.54 ± 4.41, n = 17; NMS, 88.09 ± 8.41, n = 17; **P < 0.01; Figure 5C and D). NAC treatment significantly mitigated the increase in the duration of immobility observed in both the male and female NMS groups in the FST (males: NMS group, 94.83 ± 9.19, n = 13; NMS + NAC, 44.99 ± 2.78, n = 10; females: NMS group, 88.09 ± 8.41, n = 17; NMS, 55.47 ± 6.27, n = 18; ^##^*p* < 0.01 and ^###^*p* < 0.001; Figure 5C and D). However, a decrease in the duration of climbing was observed only in the male NMS group and was alleviated by NAC treatment (Sal group, 72.27 ± 2.67, n = 10; NMS group, 46.20 ± 3.43, n=13; NMS + NAC, 79.49 ± 4.08, n = 10, *p < 0.05, ^##^*p* < 0.01; Figure 5E and F). These results indicate that NAC has a restorative effect on the depressive-like behaviors observed in both the male and female adolescent NMS groups.

### 3.6. NAC alleviated anhedonic behavior in both the male and female adolescent NMS groups

**Figure 6:**
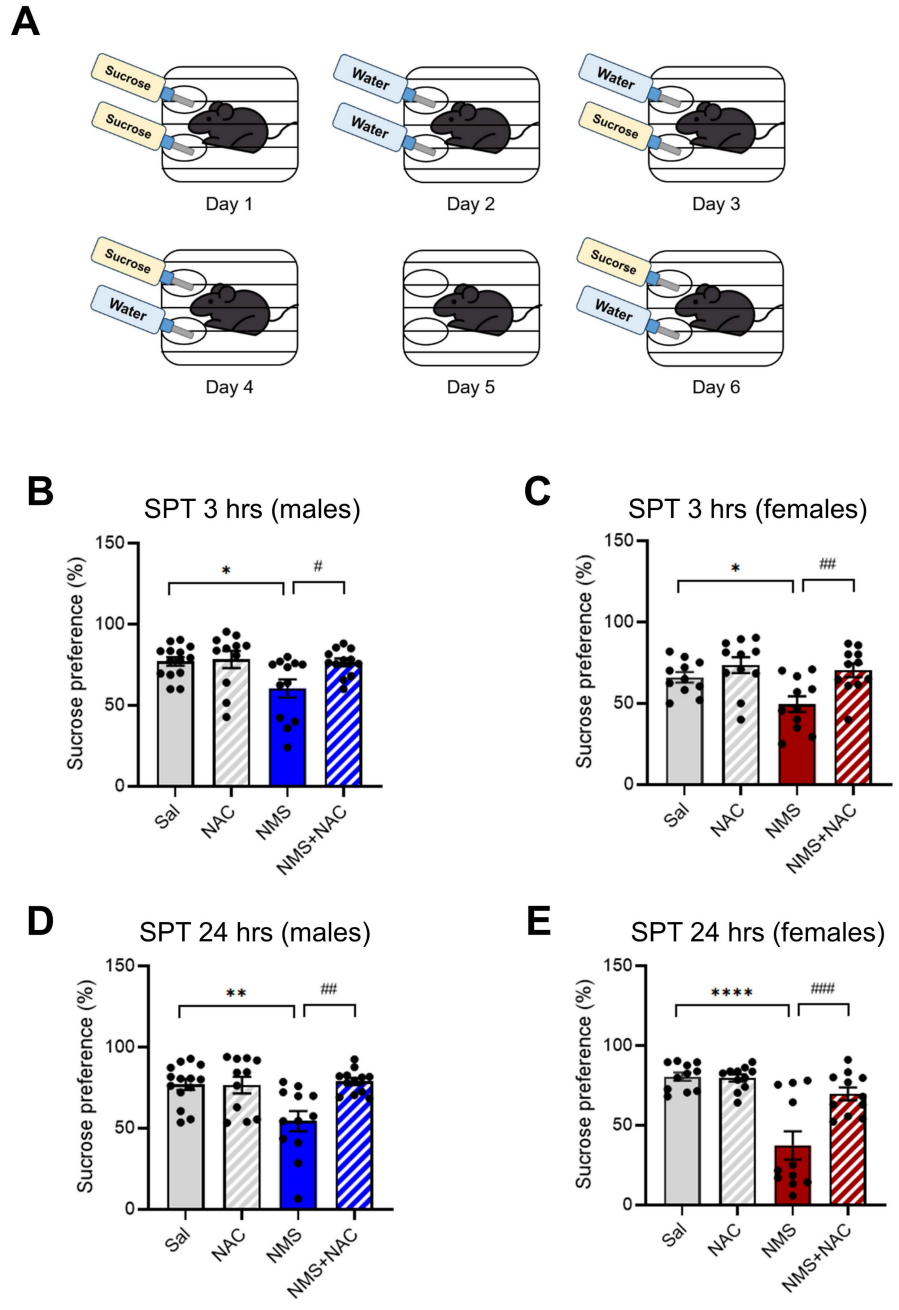
** NAC alleviates NMS-induced anhedonic behavior in male and female rats.** Adolescent NMS rats performed the SPT. (A) A schematic representation of the SPT protocol is shown. The measurement of anhedonic behavior by the SPT in adolescent male and female rats over 5 days (habituation) and 6 days (test) is presented. The bar graphs show the percentage of sucrose preference during 3 hrs (B) and 24 hrs (D) in male rats and during 3 hrs (C) and 24 hrs (E) in female rats. The data quantifying anhedonic behavior are presented as the means ± S.E.M.s. Differences in the experimental groups were determined using one-way ANOVA with Tukey's multiple comparisons test. *p < 0.05, **p < 0.01, ****p < 0.0001, ^#^*p* < 0.05, ^##^*p* < 0.01, and ^###^*p* < 0.001.

We subsequently assessed anhedonia in NMS models of depression by testing the sucrose preference (Figure 6A). Compared with the Sal group, both the male and female NMS groups presented a significant decrease in sucrose preference at both 3 hrs (males: Sal group, 76.97 ± 2.56, n = 14; NMS group, 60.38 ± 5.38, n = 12; females: Sal group, 65.98 ± 3.01, n = 11; NMS group, 49.55 ± 4.67, n = 11, *p < 0.05; Figure 6B and C) and 24 hrs (males: Sal group, 76.90 ± 3.25, n = 14; NMS group, 54.38 ± 5.95, n = 12; females: Sal group, 80.51 ± 2.44, n = 11; NMS group, 37.32 ± 8.46, n = 11, **p < 0.01 and ****p < 0.0001; Figure 6D and E). Treatment with NAC significantly mitigated the decrease in sucrose preference observed in both the male and female NMS groups at 3 hrs (males: NMS group, 60.38±5.38, n = 12; NMS+NAC group, 76.40 ± 2.33, n = 12; females: NMS, 49.55 ± 4.67, n = 11; NMS+NAC, 70.32 ± 3.93, n = 13, ^#^*p* < 0.05 and ^##^*p* < 0.01; Figure 6B and C) and 24 hrs (males: NMS, 54.38 ± 5.95, n = 12; NMS+NAC, 78.77 ± 2.07, n = 12; females: NMS, 37.32 ± 8.46, n = 11; NMS + NAC, 69.70 ± 3.80, n = 11, ^##^*p* < 0.01 and ^###^*p* < 0.001; Figure 6D and E). These results confirm that NAC resolves NMS-induced anhedonic behavior in both male and female rats.

### 3.7. NAC alleviated the reductions in antioxidant enzymes and increase in superoxide generation in the HPC of the adolescent NMS group

**Figure 7:**
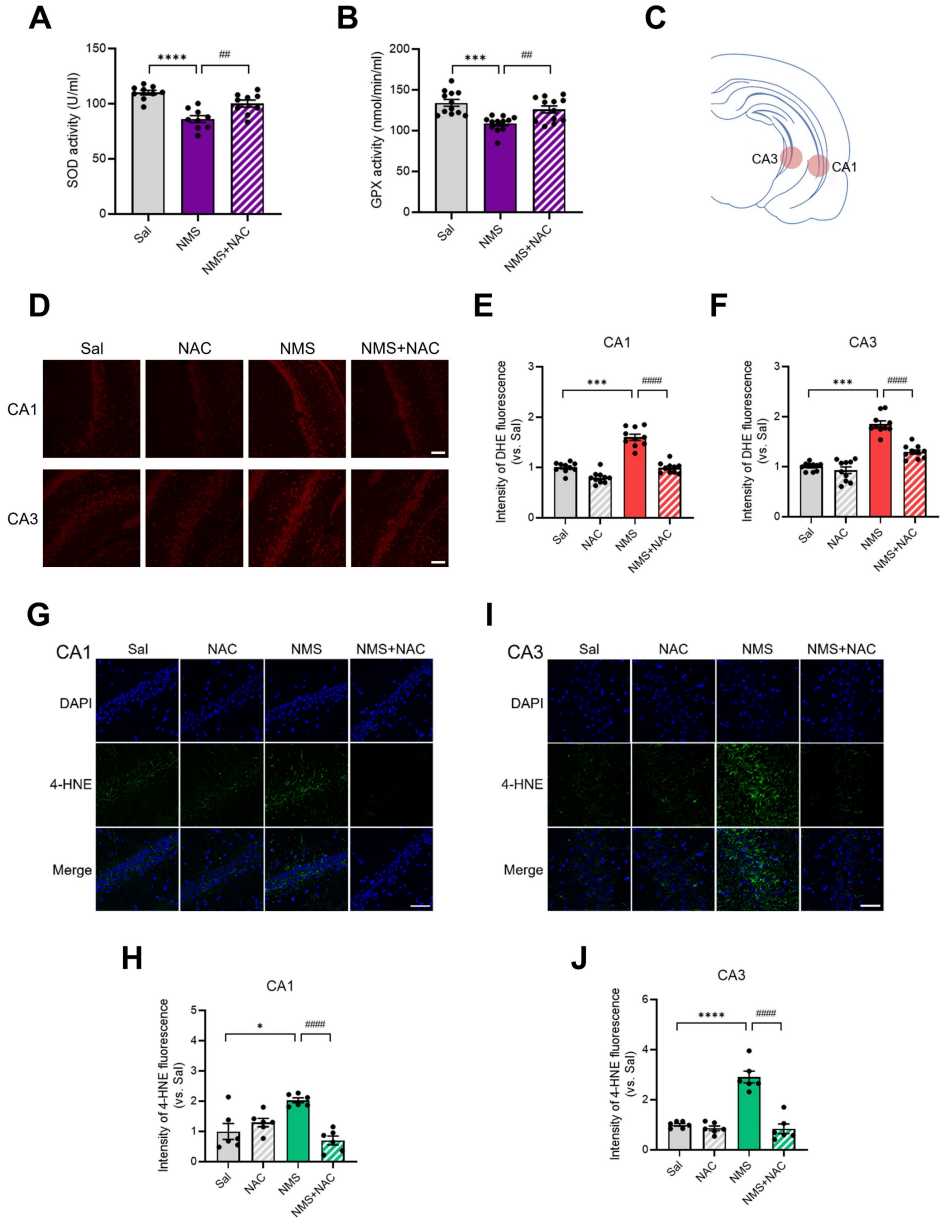
** NAC reduces oxidative stress in the vHPC, resulting in neuroprotection.** Antioxidant activities of SOD (A) and GPx (B) in the HPC of adolescent animals treated with NAC. (C) The illustration shows the stained regions of the vHPC, including the CA1 and CA3 areas. (D) Images of IF staining for DHE in the CA1 and CA3 regions of the vHPC. Scale bar, 100 μm. Quantification of DHE immunoreactivity in the CA1 (E) and CA3 (F) regions. (G and I) Sections of the vHPC (CA1 and CA3 regions) from each group of rats were stained with an anti-4-HNE antibody; scale bar, 50 μm. (H and J) Quantification of 4-HNE immunoactivity in the CA1 (G) and CA3 (I) regions. The data are presented as the means ± S.E.M.s. Differences in the experimental groups were determined using one-way ANOVA with Tukey's multiple comparisons test. *p < 0.05, ***p < 0.001, ****p < 0.0001, ^##^*p* < 0.01, and ^####^*p* < 0.0001.

We investigated whether ROS production was induced in the HPC by NMS and, if so, whether this cellular stress could be regulated by NAC. We then analyzed the activities of antioxidant enzymes and found that NMS significantly reduced the activities of SOD (Sal group, 110.06 ± 1.98, n = 9; NMS group, 86.07 ± 2.92, n=9, ****p < 0.0001; Figure 7A) and GPx (Sal group, 134.14 ± 4.00, n = 12; NMS group, 108.72 ± 2.66, n = 12, ***p < 0.001; Figure 7B) in the HPC compared with the Sal group. Treatment with NAC attenuated the NMS-induced reductions in the activities of SOD (NMS, 86.07 ± 2.92, n = 9; NMS + NAC, 100.17 ± 3.01, n = 9, ^##^*p* < 0.01; Figure 7A) and GPx (NMS, 108.72 ± 2.66, n = 12; NMS + NAC, 126.02 ± 4.08, n = 12, ^##^*p* < 0.01; Figure 7B).

We performed DHE staining to detect the amount of superoxide, a major form of ROS in the vHPC (Figure 7C-F). NMS significantly increased the superoxide level in the cornu ammonis 1 (CA1) (top panels) and CA3 regions of the vHPC (bottom panels) in adolescent animals compared with that in the Sal group (CA1: Sal group, 1.00 ± 0.03, n = 10; NMS group, 1.60 ± 0.06, n = 10; Figure 7D and E; CA3: Sal group, 1.00 ± 0.02, n = 10; NMS group, 1.85 ± 0.06, n = 10, ***p < 0.001; Figure 7D and F). NAC reduced the level of NMS-induced superoxide generation in the vHPC (CA1: NMS group, 1.60 ± 0.06, n = 10; NMS + NAC, 0.99 ± 0.03, n = 10, Figure 7D and E; CA3: NMS group, 1.85 ± 0.06, n = 10; NMS + NAC, 1.29 ± 0.04, n=10, ^####^*p* < 0.0001, Figure 7D and F).

4-HNE (4-hydroxynonenal) is a major end product of lipid peroxidation, and we performed 4-HNE staining to assess the level of oxidative stress in the vHPC. NMS significantly increased the 4-HNE level in the CA1 and CA3 regions of the vHPC during adolescence compared with that of the Sal group (CA1: Sal group, 1.00 ± 0.24, n = 6; NMS group, 2.03 ± 0.08, n = 6; Figure 7G and H; CA3: Sal group, 1.00 ± 0.05, n = 6; NMS group, 2.91 ± 0.21, n = 6; Figure 7I and J, *p < 0.05 and ****p < 0.0001). NAC reduced the level of NMS-induced lipid peroxidation in the vHPC (CA1: NMS group, 2.03 ± 0.08, n = 6; NMS+NAC, 0.70 ± 0.13, n = 6; Figure 7G and H; CA3: NMS group, 2.91 ± 0.21, n = 6; NMS + NAC, 0.84 ± 0.18, n = 6; Figure 7I and J;^ ####^*p* < 0.0001). These results clearly show that NAC rescues NMS-induced oxidative stress in the HPC of adolescent rats.

### 3.8. NAC alleviated the NMS-induced increase in iNOS expression in the HPC of adolescent animals

**Figure 8:**
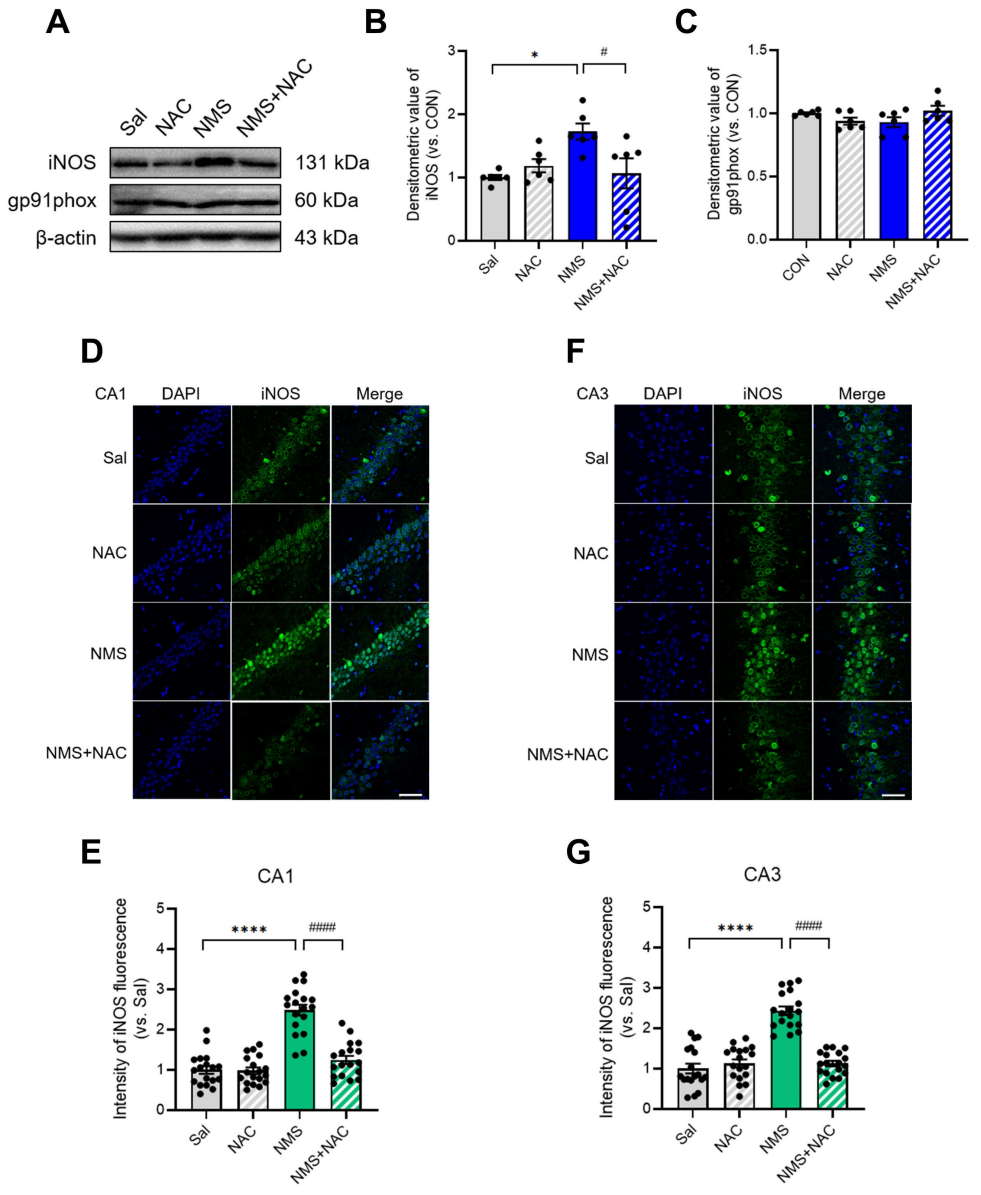
** NAC reduces iNOS levels, resulting in neuroprotection in the vHPC.** (A) Western blot analysis of iNOS and gp91^phox^ levels in the HPC of each group of rats. (B) Quantification of iNOS immunoreactivity. (C) Quantification of gp91^phox^ immunoreactivity. (D and F) Sections of the vHPC (CA1 and CA3 regions) from each group of rats were stained with an anti-iNOS antibody; scale bar, 50 μm. (E and G) Quantification of iNOS immunoreactivity in the CA1 (D) and CA3 (F) regions. The data are presented as the means ± S.E.M.s. Differences among the experimental groups were determined using one-way ANOVA with Tukey's multiple comparisons test. *p < 0.05, ****p < 0.0001, ^#^*p* < 0.05, and ^####^*p* < 0.0001.

We next examined the changes in NMS-induced inducible nitric oxide synthase (iNOS) and NADPH oxidase 2 (gp91^phox^) expression in the HPC of adolescent rats. iNOS expression was markedly increased in the HPC of NMS rats during adolescence (Sal group, 1.00 ± 0.04, n = 6; NMS group, 1.73 ± 0.12, n = 6, *p < 0.05; Figure 8A and B). However, no change in gp91^phox^ expression was observed. The injection of NAC attenuated the increase in iNOS expression induced by NMS (NMS group, 1.73 ± 0.12, n = 6; NMS+NAC group, 1.07 ± 0.22, n = 6, ^#^*p* < 0.05; Figure 8A and B). Additionally, we performed IF staining to visualize iNOS expression in the vHPC. The iNOS expression was significantly increased in the CA1 and CA3 of the vHPC in the NMS group compared with that of the Sal group (CA1: Sal group, 1.00 ± 0.09, n = 18; NMS group, 2.48 ± 0.13, n = 18; Fig. 8D and E; CA3: Sal group, 1.00 ± 0.12, n = 18; NMS, 2.43 ± 0.10, n = 18; Figure 8F and G; ****p < 0.0001). The injection of NAC reduced the increase in iNOS expression caused by NMS (CA1: NMS group, 2.48 ± 0.13, n = 18; NMS+NAC group, 1.25 ± 0.10, n = 18; Figure 8D and E; CA3: NMS group, 2.43 ± 0.10, n = 18; NMS+NAC group, 1.12 ± 0.07, n = 18; Figure 8F and G; ^####^*p* < 0.0001). These results further confirm previous findings that oxidative stress in the adolescent vHPC induced by NMS is stabilized by administering NAC.

### 3.9. NAC mitigated NMS-induced microglial activation and increased proinflammatory cytokine levels in the vHPC and prefrontal cortex

**Figure 9:**
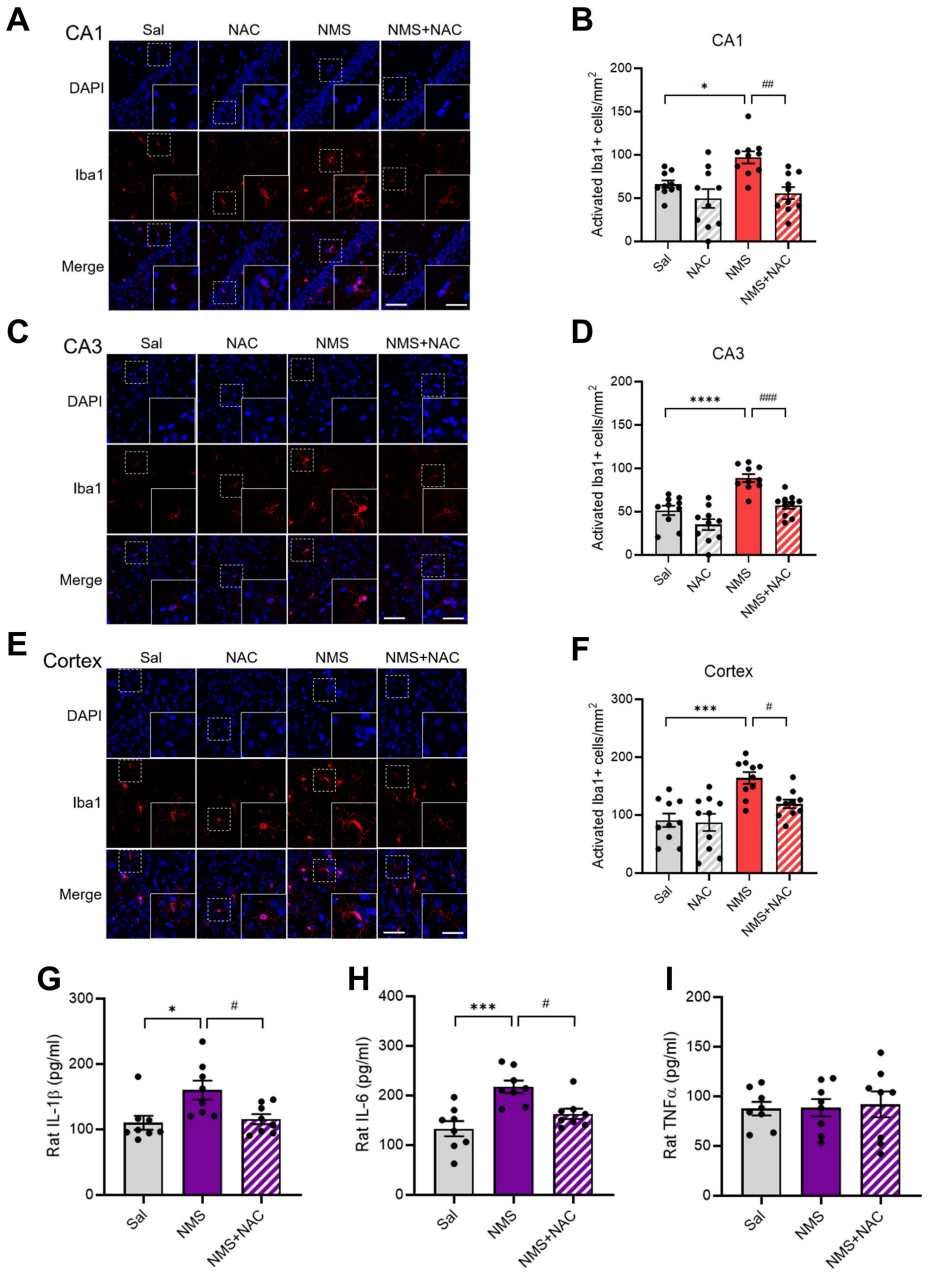
** NAC mitigates NMS-induced neuroinflammation.** Sections of the rat brain containing the vHPC were stained with an anti-Iba1 antibody. Representative images illustrate the expression of Iba1 in the CA1 (A) and CA3 (C) regions of the vHPC, as well as in the cerebral cortex (E). Scale bar, 50 μm; inset, enlarged areas. Scale bar, 25 μm. (B) Quantitative analysis of the number of activated Iba1^+^ cells/mm² in A (n=10; *p < 0.05, ^##^*p* < 0.01). (D) Quantitative analysis of the number of activated Iba1^+^ cells/mm² in C (n=10; ****p < 0.0001, ^###^*p* < 0.001). (F) Quantitative analysis of the number of activated Iba1^+^ cells/mm² in E (n=10; ***p < 0.0001, ^#^*p* < 0.05). ELISAs of cytokine levels in the hippocampus of adolescent animals treated with NAC. (G) Quantitative analysis of the IL-1β concentration (n=8; *p < 0.05 and ^#^*p* < 0.05). (H) Quantitative analysis of the IL-6 concentration (n=8; ***p < 0.001 and ^#^*p* < 0.05). (I) Quantitative analysis of the TNFα concentration (n=8; *p* > 0.05). The bar graph shows the quantification of the data, which are presented as the means ± S.E.M.s. Differences among the experimental groups were determined using one-way ANOVA with Tukey's multiple comparisons test.

We evaluated whether NMS induced the activation of astrocytes and microglia and whether this cellular stress could be mitigated by NAC. We conducted IF staining for GFAP in the CA1 and CA3 regions of the vHPC but did not observe significant differences among the groups (Supplementary Figure S1A-D). Reactive microglia are reported to have larger soma sizes and thicker, less branched processes 30. We set the criteria for activated microglia as having a cell area greater than 200 μm^2^ 30, 31 and measured their numbers for analysis. Compared with the Sal group, the NMS group presented significantly greater activation of microglia in the CA1 and CA3 regions of the vHPC and cortex (CA1: Sal group, 66.12 ± 4.02; NMS group, 97.11 ± 6.62; Figure 9A and B; CA3: Sal group, 51.45 ± 5.12; NMS group, 88.84 ± 4.28; Figure 9C and D; cortex: Sal group, 90.91 ± 11.04; NMS group, 164.05 ± 9.59; Figure 9E and F; n = 10, *p < 0.05, ***p < 0.001, and ****p < 0.0001). NAC alleviated the increase in the number of Iba1^+^ microglia in the CA1 and CA3 regions of the vHPC and cortex induced by NMS (CA1: NMS group, 97.11 ± 6.62; NMS+NAC group, 55.79 ± 6.57; Figure 9A and B; CA3: NMS group, 88.84 ± 4.28; NMS+NAC group, 57.44 ± 3.77; Figure 9C and D; cortex: NMS group, 164.05 ± 9.59; NMS+NAC group, 119 ± 7.07; Figure 9E and F; n = 10, ^#^*p* < 0.05, ^##^*p* < 0.01, and ^###^*p* < 0.001). Next, we assessed the levels of proinflammatory cytokines, including IL-1β, IL-6, and TNFα, using ELISAs. Significant increases in IL-1β and IL-6 levels, but not TNF levels, were detected in the prefrontal cortex of adolescent rats from the NMS group (IL-1β:Sal group, 110.12 ± 10.05; NMS group, 160.13 ± 13.62; Figure 9G; IL-6:Sal group, 133.15 ± 14.26; NMS group, 217.65 ± 11.65; Figure 9H; n = 8; *p < 0.05 and ***p < 0.001). Notably, the NAC injection attenuated the increased levels of IL-1β and IL-6 induced by NMS (IL-1β: NMS group, 160.13 ± 13.62; NMS + NAC group, 115.62 ± 7.31; Figure 9G; IL-6: NMS group, 217.65 ± 11.65; NMS+NAC, 162.90 ± 9.81; Figure 9H; n = 8, ^#^*p* < 0.05).

### 3.10. NAC reversed the NMS-induced downregulation of EAAC1 expression in the HPC

**Figure 10:**
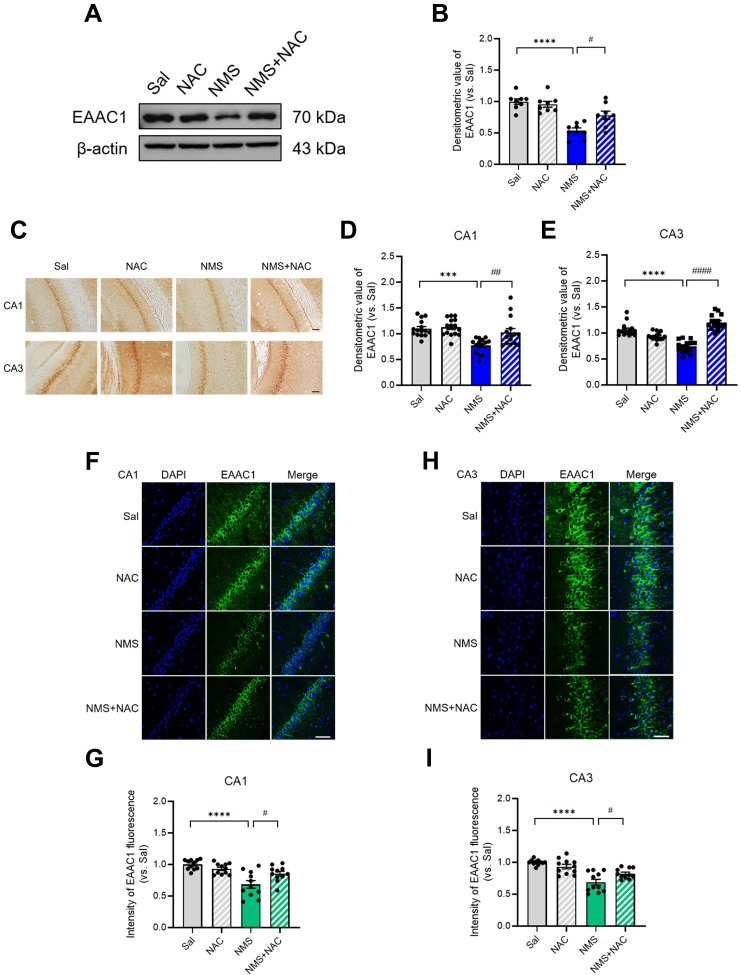
** NAC reverses the NMS-induced downregulation of EAAC1 expression in the vHPC.** (A) Western blot analysis of EAAC1 levels in the HPC of each group of rats. (B) Quantitative analysis of EAAC1 immunoreactivity in (A). (C) Sections of the vHPC (CA1 and CA3 regions) from each group of rats were stained with an anti-EAAC1 antibody. Scale bar, 50 μm. Quantitative analysis of EAAC1 immunoreactivity in the CA1 (D) and CA3 (E) regions. (F and H) Sections of the rat brain containing the vHPC were stained with an anti-EAAC1 antibody. Scale bar, 50 μm. (G and I) Bar graphs display the results of the quantitative analysis presented as the means ± S.E.M.s. Differences in the experimental groups were determined using one-way ANOVA with Tukey's multiple comparisons test. ***p < 0.001, ****p < 0.0001, ^#^*p* < 0.05, ^##^*p* < 0.01, and ^####^*p* < 0.0001.

We examined whether NAC influences the change in EAAC1 expression induced by NMS in the HPC during adolescence. We observed a significant decrease in EAAC1 expression in the HPC of NMS rats (Sal group, 1.00 ± 0.04; NMS group, 0.53 ± 0.04; n = 8; ****p < 0.0001; Figure 10A and B). However, the NAC injection reversed the NMS-induced decrease in EAAC1 protein expression (NMS group, 0.53 ± 0.04; NMS+NMS group, 0.78 ± 0.06; n = 8; ^#^*p* < 0.05; Figure 10A and B). Furthermore, we investigated the EAAC1 expression induced by NMS in the vHPC of adolescent brain tissue slices by immunostaining. We observed a significant reduction in EAAC1 protein expression in the CA1 and CA3 regions of the vHPC in NMS rats (CA1: Sal group, 1.00 ± 0.04; NMS group, 0.78 ± 0.04; Figure 10C and D; CA3: Sal group, 1.00 ± 0.03; NMS, 0.74 ± 0.03; Figure 10C and E; n = 15, ***p < 0.001 and ****p < 0.0001). The NAC injection alleviated the NMS-induced decrease in EAAC1 expression (CA1: NMS group, 0.78 ± 0.04; NMS+NAC group, 1.03 ± 0.07; Figure 10C and D; CA3: NMS group, 0.74 ± 0.03; NMS+NAC group, 1.20 ± 0.03; Figure 10C and E; n = 15, ^##^*p* < 0.01 and ^####^*p* < 0.0001). We confirmed these results using IF staining and observed similar outcomes (Figure 10F-I). These findings suggest that NAC can reverse the NMS-induced downregulation of EAAC1 expression in the vHPC.

### 3.11. NAC alleviated depressive-like behaviors in adolescent EAAC1^-/-^ mice

**Figure 11:**
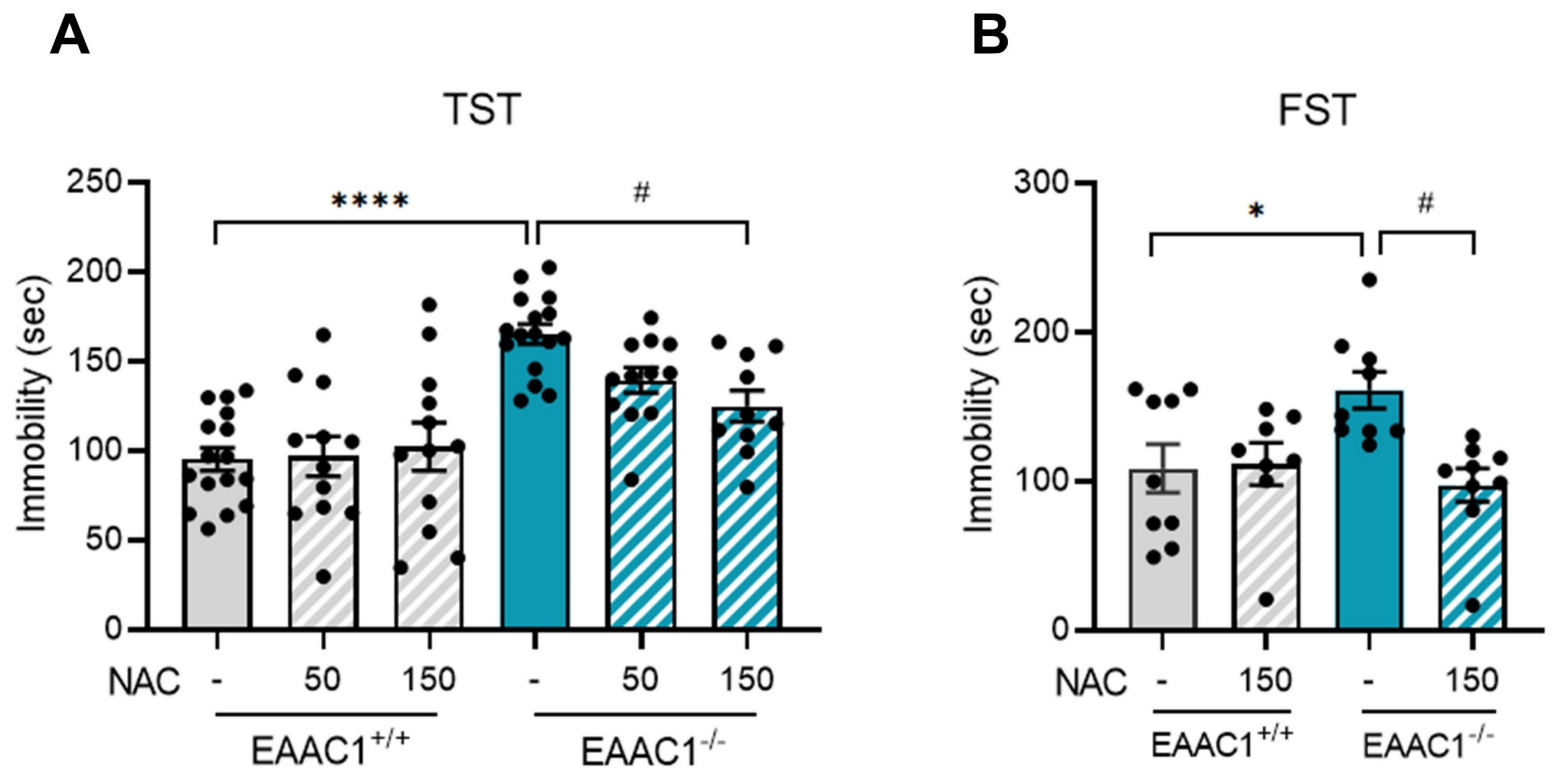
** NAC mitigates depressive-like behaviors in adolescent EAAC1^-/-^ mice.** The mice were subjected to the FST and TST. The bar graphs show the immobility time of the mice during the TST (A) and FST (B). The data quantifying depressive-like behaviors are presented as the means ± S.E.M.s. Differences in the experimental groups were determined using one-way ANOVA with Tukey's multiple comparisons test. *p<0.05, ****p<0.0001, and ^#^*p* < 0.05.

We assessed the effects of NAC on depressive-like behavior in adolescent EAAC1^-/-^ mice using the TST. Compared with EAAC1^+/+^ mice, EAAC1^-/-^ mice exhibited a significant increase in immobility time (EAAC1^+/+^ mice, 95.23 ± 6.18, n=16; EAAC1^-/-^ mice, 165.14 ± 5.32, n = 16; ****p < 0.0001; Figure 11A). Although 50 mg/kg NAC, which improved depression-like behaviors induced by NMS, slightly reduced depressive symptoms in EAAC1^-/-^ mice, the difference was not significant. Therefore, we increased the concentration to 150 mg/kg for further comparison. At this concentration, NAC significantly reduced the increase in immobility time in EAAC1^-/-^ mice (EAAC1^-/-^ mice, 165.14 ± 5.32, n=16; EAAC1^-/-^ mice + NAC, 124.89 ± 8.22, n = 10, ^#^*p* < 0.05; Figure 11A). We performed the FST using adolescent EAAC1^-/-^ mice to further confirm these findings. Similar to the TST results, the immobility time of the EAAC1^-/-^ mice was significantly increased compared to that of EAAC1^+/+^ mice (EAAC1^+/+^ mice, 108.64 ± 15.29, n = 9; EAAC1^-/-^ mice, 161.20 ± 11.51, n = 9, *p < 0.05; Figure 11B). Treatment with 150 mg/kg NAC also mitigated the increase in the immobility time of EAAC1^-/-^ mice (EAAC1^-/-^ mice, 161.20 ± 11.51, n = 9; EAAC1^-/-^ mice + NAC, 97.42 ± 10.53, n = 9, ^#^*p* < 0.05; Figure 11B). These results suggest that NAC can alleviate depressive-like behaviors in adolescent EAAC1^-/-^ mice.

### 3.12. NAC alleviated anhedonic behavior in adolescent EAAC1^-/-^ mice

**Figure 12:**
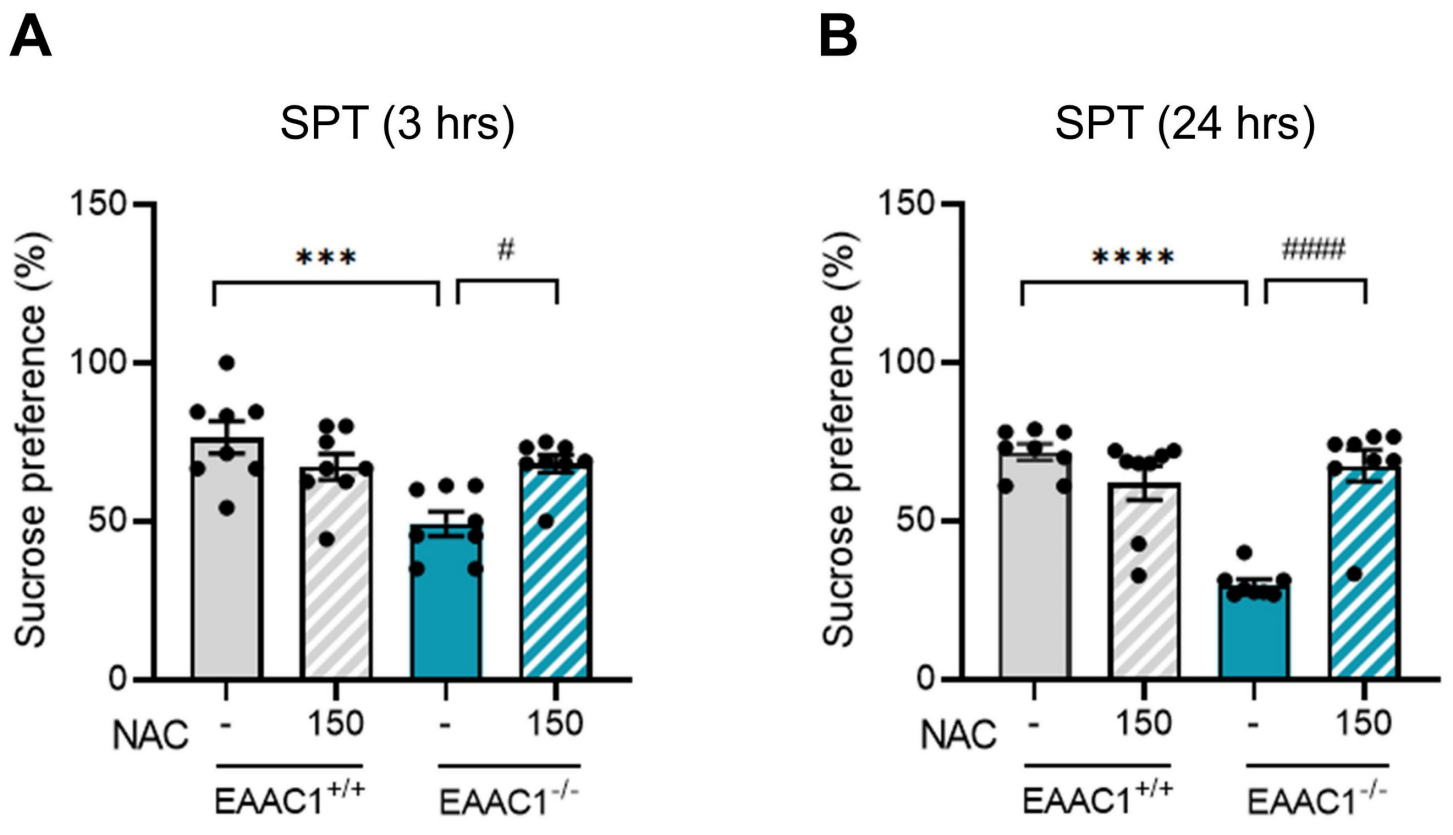
** NAC attenuates anhedonic behavior in adolescent EAAC1^-/-^ mice.** Adolescent EAAC1^+/+^ and EAAC1^-/-^ mice were subjected to the SPT. The bar graph shows the % sucrose preference of male mice at 3 hrs (A) and 24 hrs (B). The data quantifying anhedonic behavior are presented as the means ± S.E.M.s. Differences in the experimental groups were determined using one-way ANOVA with Tukey's multiple comparisons test. ***p<0.001, ****p<0.0001, ^#^*p*<0.05, and ^####^*p*<0.0001.

We evaluated the effects of NAC on anhedonic behavior in adolescent EAAC1^-/-^ mice using the SPT. Compared with EAAC1^+/+^ mice, EAAC1^-/-^ mice presented a significant decrease in sucrose preference at both 3 hrs (EAAC1^+/+^ mice, 76.46 ± 4.75, n = 8; EAAC1^-/-^ mice, 49.19±3.62, n = 8, ***p < 0.001; Figure 12A) and 24 hrs (EAAC1^+/+^ mice, 71.69 ± 2.39, n = 8; EAAC1^-/-^ mice, 29.98 ± 1.47, n = 8, ****p < 0.0001; Figure 12B). However, NAC significantly alleviated the decrease in sucrose preference in adolescent EAAC1^-/-^ mice both at 3 hrs (EAAC1^-/-^ mice, 49.19 ± 3.62, n = 8; EAAC1^-/-^ mice + NAC, 68.23 ± 2.59, n = 8, ^#^*p* < 0.05; Figure 12A) and at 24 hrs (EAAC1^-/-^ mice, 29.98 ± 1.47, n = 8; EAAC1^-/-^ mice + NAC, 67.45 ± 4.72, n = 8, ^####^*p* < 0.0001; Figure 12B). These results suggest that NAC can alleviate anhedonic behavior in EAAC1^-/-^ mice.

### 3.13. NAC did not rescue impulsive-like behaviors in EAAC1^-/-^ mice

**Figure 13:**
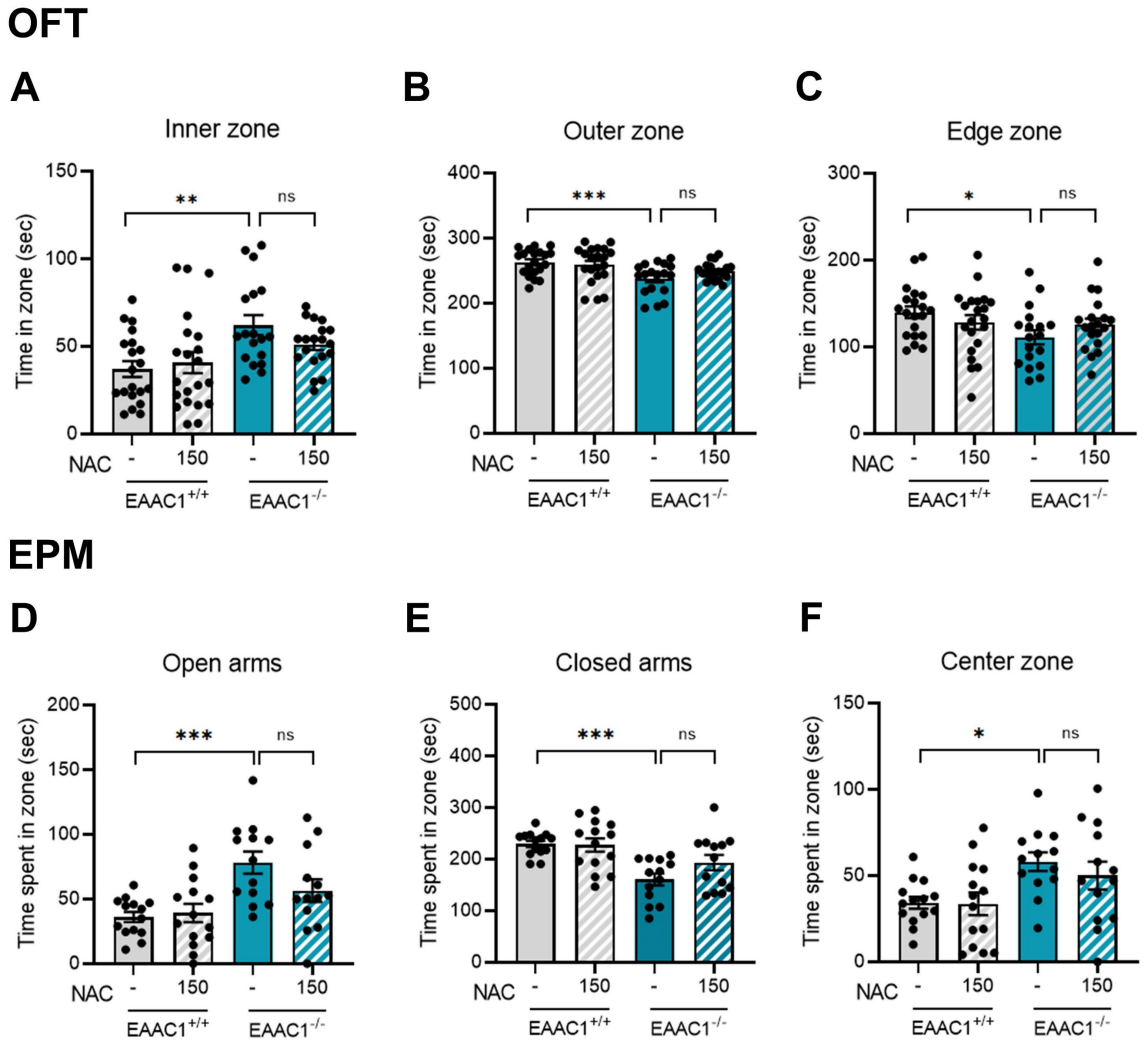
** Effects of NAC on impulsive behaviors in adolescent EAAC1**^-/-^** mice in the OFT and EPM.** The bar graph represents the time spent in the inner zone (A), outer zone (B), and edge zone (C) of the OFT. The data also show the time spent in the open arms (D), closed arms (E), and center zone (F) of the EPM. The data are presented as the means ± S.E.M.s. Differences in the experimental groups were determined using one-way ANOVA with Tukey's multiple comparisons test. *p < 0.05, **p < 0.01, and ***p < 0.001.

We investigated whether NAC could alleviate the impulsive behaviors observed in EAAC1^+/+^ mice. Adolescent mice performed to OFT to assess the effects of NAC on impulsive behaviors. The time spent in the inner zone by the EAAC1^-/-^ mice was significantly longer than that of EAAC1^+/+^ mice (EAAC1^+/+^ mice, 37.07 ± 4.34, n = 20; EAAC1^-/-^ mice, 62.12 ± 5.56, n = 18, **p < 0.01; Figure 13A). However, NAC did not reverse the increased time spent by the EAAC1^-/-^ mice in the inner zone. The time spent in the outer and edge zones by the EAAC1^-/-^ mice was also significantly shorter than that spent by the EAAC1^+/+^ mice (outer zone: EAAC1^+/+^ mice, 262.93 ± 4.34, n=20; EAAC1^-/-^ mice, 237.88 ± 12.24, n = 18, ***p < 0.001; Figure 13B; edge zone: EAAC1^+/+^ mice, 139.96 ± 6.60, n = 20; EAAC1^-/-^ mice, 111.01 ± 7.91, n = 18, *p < 0.05; Figure 13C). NAC did not restore the decreased time spent by the EAAC1^-/-^ mice in the outer and edge zones.

The effects of NAC on impulsive behaviors in adolescent EAAC1^-/-^ mice were further evaluated using the EPM test. We observed an increase in the time spent in the open arms and center zone by EAAC1^-/-^ mice (open arms: EAAC1^+/+^ mice, 36.15 ± 3.77, n=14; EAAC1^-/-^ mice, 78.17 ± 8.26, n = 13, Figure 13D; center zone: EAAC1^+/+^ mice, 34.24 ± 3.33, n = 14; EAAC1^-/-^ mice, 58.10 ± 5.18, n = 13, Figure 13F; *p < 0.05 and ***p < 0.001). In contrast, EAAC1^-/-^ mice spent less time in the closed arms (closed arms: EAAC1^+/+^ mice, 229.61 ± 5.92, n = 14; EAAC1^-/-^ mice, 160.52 ± 11.12, n = 13, ***p < 0.001; Figure 13E). However, NAC did not reverse the increased time spent in the open arms and center zone or the decreased time spent in the closed zone by the EAAC1^-/-^ mice. These results suggest that the impulsive behaviors observed in adolescent EAAC1^-/-^ mice are not mitigated by the NAC injection.

## 4. Discussion

ELS has been associated with psychopathology later in life. Psychosocial risk factors include childhood neglect or abuse, relationship stressors, and states of mind such as hopelessness 32. Early-life stress has been linked to a wide range of negative effects on numerous neuronal systems, the understanding of which remains incomplete. In our study, we utilized NMS as a model of ELS. This model involves separating pups from their mothers and siblings for 3 hrs daily during the first 3 weeks of life.

We previously discovered that the expression of the EAAC1 protein was significantly reduced in the hippocampus and cerebral cortex of adolescent NMS model rats. EAAC1, while broadly distributed throughout the brain, is the primary transporter for excitatory amino acids in neurons 33. The glial cell glutamate transporters GLAST and GLT1 primarily facilitate synaptic glutamate clearance, whereas EAAC1 is more involved in cysteine uptake than in glutamate clearance in the brain 34. EAAC1 plays crucial, albeit only partially characterized, roles in the regulation of glutamatergic synapses and in the preservation of neuronal integrity 35. Our findings indicate that adolescent NMS model rats of both sexes exhibit impulsive and depressive-like behaviors. In this study, we aimed to determine whether the observed behavioral changes in the NMS model rats were related to a decrease in cysteine uptake function due to reduced EAAC1 protein expression. Cysteine has the lowest intracellular concentration, which limits the rate of GSH synthesis in the presence of oxidative stress. NAC can cross the plasma membrane of cells and is rapidly hydrolyzed to cysteine, which can be incorporated into GSH 36. NAC is potentially useful in the treatment of various psychiatric disorders 37. Although not conclusive, recent clinical data suggest that NAC may be a useful adjunct medication for treating addiction and substance abuse, schizophrenia, obsessive‒compulsive and related disorders, and mood disorders 37.

We initially observed the effect of NAC on depressive-like behaviors induced by NMS in both adolescent male and female rats. Adolescent depression is a prevalent disorder with significant lifetime morbidity and mortality. Numerous psychosocial and biological risk factors for adolescents to develop depression have been identified. Compared with a placebo, NAC was shown to significantly alleviate depressive symptoms and improve functionality in adult patients 38, 39. Another clinical study reported that although the results of NAC treatment for depression disorder remain mixed and additional evidence is needed, the authors considered NAC a promising treatment option for mood disorders 40. Treatment with NAC for 3 days corrected the depressive-like phenotype in adult mice by inducing resilience to stress through an increase in xCT expression 41. In the TST of adult mice, the antidepressant effects of NAC are blocked by AMPA antagonists 42. We found that both male and female NMS rats had increased durations of immobility in the TST and FST. These depressive-like behaviors were reversed by NAC injection in both male and female NMS rats during adolescence. Approximately 11% of adolescents report experiencing depression 43. According to a recent report, chronic use of cannabis is associated with depression 44, and NAC, an over-the-counter medication thought to regulate glutamate transmission and reduce oxidative stress, has produced promising outcomes in both enhancing the efficacy of abstinence-based cannabis treatment programs 45 and reducing depressive symptoms 38. Furthermore, we observed that male adolescent EAAC1^-/-^ mice exhibited depressive-like behaviors similar to those of NMS rats in the TST and FST. These depressive-like behaviors were reversed by the NAC injection in male adolescent EAAC1^-/-^ mice. These results indicate that a reduction in the cysteine uptake function of EAAC1 is important for causing depressive-like behavior.

We conducted a sucrose preference test on a separate group of NMS rats to further evaluate the impact of NAC on depressive-like behaviors. We discovered that both male and female adolescent NMS rats presented a significant decrease in sucrose preference, which indicates anhedonic behavior. This anhedonic behavior was reversed by the NAC injection in both male and female adolescent NMS rats. We also found that male adolescent EAAC1^-/-^ mice exhibited anhedonic behavior, similar to NMS rats, as evidenced by the TST and FST. These anhedonic behaviors were reversed by the NAC injection in male adolescent EAAC1^-/-^ mice. These results suggest that the cysteine uptake function of EAAC1 is also related to anhedonic behavior, a phenotype of depression. Taken together, these findings demonstrate that NAC can alleviate depression in adolescents.

Next, we observed increased impulsive-like behaviors in both male and female NMS rats during adolescence. We confirmed that NMS increased impulsive-like behaviors in adolescent animals of both sexes, as both male and female NMS rats spent more time in the inner zone of the OFT and in the open arms of the EPM. Staying in the inner zone of the OFT and the open arms of the EPM is typically as assumed to indicate anxiolytic effects but is also considered more impulsive than in the control group 28, 46, 47. The effects of NMS on behaviors in the OFT and EPM vary (increase, decrease, or no effect) depending on the protocols, such as strains, individual or sibling separation, separation time or duration, and sample size 48, 49. Thus, we confirmed impulsive behaviors in the NMS model rats by performing additional tests. Impulsive behavior is a core symptom of psychiatric disorders, including ADHD, but animal behavioral techniques to evaluate impulsivity remain limited. The cliff avoidance test (CAT) is a common method for assessing impulsive behavior 20, 21. In the CAT, no significant difference was observed in the fall rate to the floor area within the 10-minute evaluation, but both sexes spent more time in dangerous areas (boundary + floor area). In the visual cliff avoidance test (VCAT), a significant increase in cliff steps was observed in both male and female NMS rats. Although the VCAT is used to assess vision development in infancy 50, it is also effective for evaluating impulsivity in adolescence and adulthood 23, 51. Based on the CAT results, additional research is needed to determine whether the increased measurement time reveals significant differences in fall rates, similar to VCAT findings.

Research has suggested that artificially reared rats are more impulsive; however, this effect can be reduced by maternal licking-like stimulation 52. Deprivation of the mother for 24 hrs on PND 3 led to a significant increase in impulsive actions 53. Furthermore, we confirmed that adolescent male EAAC1^-/-^ mice exhibited impulsive-like behaviors similar to those of NMS rats, as evidenced by the results from the OFT and EPM. EAAC1^-/-^ mice spent significantly more time in the inner zone of the OFT and in the open arms of the EPM.

Interestingly, NAC treatment notably ameliorated impulsive-like behaviors exclusively in male NMS rats and not in female NMS rats. This pattern was consistently observed across various behavioral analyses related to impulsivity, including the OFT, EPM, CAT, and VCAT. The brains, physiology, and immune systems of males and females differ in numerous ways 54, 55. These differences reflect the distinct and specialized roles that males and females play in ensuring reproductive success. Structural and hormonal variations contribute to notable differences in how males and females respond to stress, injury, and medications 54. Importantly, females with ADHD, both girls and women, often display higher rates of impairment and comorbidity than boys and men do, starting in adolescence and continuing into adulthood. Girls diagnosed with ADHD face an increased risk of significant health outcomes, including increased suicide attempts, mood disorders, risky sexual behavior, substance use, and binge drinking 56-58. Despite the high prevalence of depressive symptoms among young people with ADHD, few studies have investigated why these symptoms intensify as these individuals age. However, recent studies suggest that these effects increase as young people progress through puberty, particularly in girls, indicating potential hormonal influences 59. In addition, we explored the impact of NAC on impulsive behaviors of EAAC1^-/-^ mice. In contrast to our expectations, the impulsive behaviors observed in male EAAC1^-/-^ mice in both the OFT and EPM tests were slightly improved following the NAC injection, but the effects were not significant. These findings suggest that NAC did not effectively mitigate impulsive behaviors in male EAAC1^-/-^ mice.

Based on the observation of the increased amelioration of impulsive and depressive-like behaviors induced by NMS following treatment with NAC, biochemical changes in the vHPC and prefrontal cortex were observed. Oxidative stress is a hallmark of pathological conditions with prolonged or intense pro-oxidative responses that produce structural and functional cellular alterations. A study of the impact of MS (maternal separation) across stages of the lifespan (adolescent, adult, and aged) on mitochondrial activity in peripheral blood mononuclear cells in rats revealed a disrupted cell cycle, long-term increases in mitochondrial activity, and increased sensitivity to H_2_O_2_-induced oxidative stress in adolescent and adult rats *in vitro* 60. Another study of the effects of MS on the oxidative status of adult mice revealed increased plasma levels of thiobarbituric reactive substances (TBARS) and decreased catalase activity in the hippocampus 61. In male rats and mice, MS has also been shown to be associated with increased ROS and mitochondrial glutathione levels in the cardiac tissue of these animals 62. Another ELS study using an animal model of early SI in rats revealed decreased activity, but increased levels of hydrogen peroxide in certain brain regions, of which prefrontal cortex and hippocampus were the most vulnerable 63. In this study, we confirmed that NMS significantly increased superoxide levels in the CA1 and CA3 regions of the vHPC during adolescence. Moreover, NAC reduced the level of NMS-induced superoxide generation. In another animal model, ELS induced by MCD was associated with increased protein carbonyl levels and decreased SOD and catalase activities in the brain in the early postnatal phase 64, whereas other studies also revealed similar long-term alterations in basal antioxidant defenses following ELS 65. We further confirmed that NMS reduced the activities of SOD and GPx in the HPC of adolescent rats. The NAC injection reversed the NMS-induced reductions in the activities of SOD and GPx.

A recent report indicated that MS with early weaning led to an increase in aortic superoxide production and endothelial dysfunction. These changes were due to elevated expression of the NADPH oxidase subunits NOX2 and NOX4 in adult animals 66. The gp91^phox^, which is the catalytic core of NADPH oxidase (NOX) and a biomarker of NOX activation, is acknowledged as a measure of systemic oxidative stress 67, 68. However, these changes were not observed in the HPC of adolescent rats subjected to NMS. On the other hand, a significant increase in iNOS expression was noted in the HPC of adolescent NMS rats. Immunostaining confirmed that iNOS expression was elevated in the CA1 and CA3 regions of the vHPC. This increase in iNOS expression was significantly reduced in the group treated with NAC. Nitric oxide synthases (NOSs) are a group of enzymes that catalyze the production of nitric oxide (NO) from L-arginine. The inducible isoform, iNOS, plays a role in the immune response and is induced by cytokines, oxidative stress, and inflammatory reactions to produce NO 69, 70. We examined the activity of astrocytes and microglia to identify inflammatory reactions. While no increase in astrocyte activity was detected in the CA1 and CA3 regions of the vHPC, microglial activity was significantly elevated not only in the vHPC but also in the cortex. This increase in microglial activity was significantly reduced in both the vHPC (CA1 and CA3) and cortex of the group treated with NAC. Furthermore, we discovered that the levels of IL-1β and IL-6, but not TNFα, were significantly elevated in the prefrontal cortex of adolescent NMS rats. The administration of NAC reduced the increases in IL-1β and IL-6 levels, and these results confirmed that early-life stress caused by NMS increases inflammation in the brain tissue of adolescent animals and that the injection of NAC ameliorates this inflammatory response.

Previously, we reported that EAAC1 expression was markedly decreased in the vHPC and cortex of NMS rats 16. Therefore, we investigated whether the recovery effect of NAC on NMS rats altered the expression of EAAC1, and we confirmed that the decreased EAAC1 expression in the NMS group was significantly reversed by the NAC injection.

The recovery of the reduced expression of the EAAC1 protein observed in NMS rats administered NAC may explain why the previously observed impulsive behavior in NMS rats was reversed in the group injected with NAC but not in the EAAC1^-/-^ mice. Furthermore, the depressive-like behaviors observed in both NMS rats and EAAC1^-/-^ mice were reversed by the NAC injection, suggesting that the ability of EAAC1 to transport cysteine into cells may be important in the mechanism of depression. Although additional research is needed, the impulsive-like behavior may involve the glutamate reuptake function of EAAC1 rather than the cysteine uptake function.

## 5. Conclusions

Our study underscores the long-lasting effects of early-life stress on depressive behaviors. These findings highlight the potential therapeutic effect of NAC on mitigating depression. We also delved into the role of EAAC1 in the vHPC in response to stress. Our research further investigated the biochemical changes induced by stress in various brain regions. A key aspect of our study is the exploration of neuroinflammatory responses in animal models of stress. These findings collectively improve our understanding of the complex interplay between early-life stress, depression, and neuroinflammation and open new avenues for potential therapeutic interventions.

## Supplementary Material

Supplementary figure.

## Abbreviations

4-HNE: 4-Hydroxynonenal
CA: Cornu ammonis
CAT: Cliff avoidance test
CNS: Central nervous system
DHE: Dihydroethidium
dHPC: Dorsal hippocampus
EAAC1: Excitatory amino acid carrier 1
EAATs: Excitatory amino acid transporters
ELS: Early life stress
EPM: Elevated plus maze
FST: Forced swimming test
GP91^phox^: NADPH oxidase 2
GPx: Glutathione peroxidase
GSH: Glutathione
HPC: Hippocampus
iNOS: Inducible nitric oxide synthase
KO: knockout
MS: Maternal separation
NAC: N-Acetylcysteine
NMS: Neonatal maternal separation
NO: Nitric oxide
OFT: Open field test
PND: Postnatal day
ROS: Reactive oxidative stress
SI: Social isolation
SPT: Sucrose preference test
TST: Tail suspension test
VCAT: visual cliff avoidance test
vHPC: Ventral hippocampus
