# Supplementary material for: N-Acetylcysteine Alleviates Depressive-Like Behaviors in Adolescent EAAC1-/- Mice and Early Life Stress Model Rats
Source: Int J Biol Sci. 2024 Oct 7;20(14):5450–73. doi: 10.7150/ijbs.97723 (PMC11528454; doi:10.7150/ijbs.97723)
Supplement: Supplementary file 1 — Supplementary figure. [file ijbsv20p5450s1.pdf]

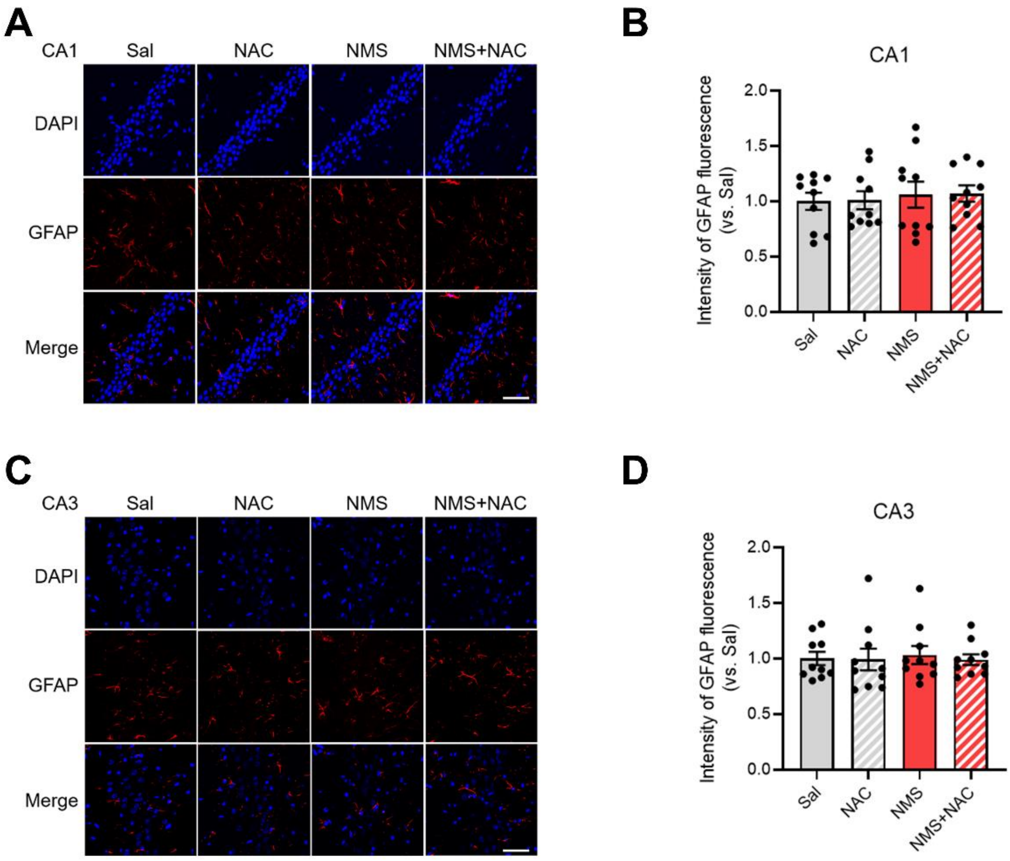

Supplementary Figure 1. NMS did not change activation of astrocytes

Section of rat brain were stained with anti-GFAP in vHPC. Representative images show the expression of GFAP in the CA1 (A) and CA3 (C) regions of the vHPC. Scale bar, 50 μm. (B) Quantification analysis of GFAP immunoreactivity in A (n = 10; $p > 0.05$). (D) Quantification analysis of GFAP immunoreactivity in C (n = 10; $p > 0.05$). Bar graph displaying the quantification analysis of expressed as the means ± S.E.M. Differences in the experimental groups were determined by one-way ANOVA with Tukey's multiple comparisons test.
